# Age-Varying Susceptibility to the Delta Variant (B.1.617.2) of SARS-CoV-2

**DOI:** 10.1001/jamanetworkopen.2022.3064

**Published:** 2022-03-18

**Authors:** June Young Chun, Hwichang Jeong, Yongdai Kim

**Affiliations:** 1 Department of Internal Medicine, National Cancer Center, Goyang, South Korea; 2 Department of Statistics, Seoul National University, Seoul, South Korea

## Abstract

**Importance:**

The Delta variant (B.1.617.2) is estimated to be more transmissible than previous strains of SARS-CoV-2, especially among children and adolescents. However, to our knowledge, there are no reports confirming this to date.

**Objective:**

To gain a better understanding of the association of age with susceptibility to the Delta variant of SARS-CoV-2.

**Design, Setting, and Participants:**

This decision analytic model used an age-structured compartmental model using the terms symptom onset (*S*), exposure (*E*), infectious (*I*), and quarantine (*Q*) (SEIQ) to estimate the age-specific force of infection, combining age-specific contact matrices and observed distribution of periods between each stage of infection (*E* to *I* [ie, latent period], *I* given *S*, and *S* to *Q* [ie, diagnostic delay]) developed in a previous contact tracing study. A bayesian inference method was used to estimate the age-specific force of infection (*S* to *E*) and, accordingly, age-specific susceptibility. The age-specific susceptibility during the third wave (ie, before Delta, from October 15 to December 22, 2020, when the COVID-19 vaccination campaign was not yet launched) and the fourth wave (ie, the Delta-driven wave, from June 27 to August 21, 2021) in Korea were compared. As vaccine uptake increased, individuals who were vaccinated were excluded from the susceptible population in accordance with vaccine effectiveness against the Delta variant. This nationwide epidemiologic study included individuals who were diagnosed with COVID-19 during the study period in Korea. Data were analyzed from September to November 2021.

**Exposures:**

Age group during the third wave (ie, before Delta) and fourth wave (ie, Delta-driven) of the COVID-19 pandemic in South Korea.

**Main Outcomes and Measures:**

Age-specific susceptibility during the third and fourth waves was estimated.

**Results:**

Among 106 866 confirmed COVID-19 infections (including 26 597 infections and 80 269 infections during the third and fourth waves of COVID-19 in Korea, respectively), a significant difference in age-specific susceptibility to the Delta vs pre-Delta variant was found in the younger age group. After adjustment for contact pattern and vaccination status, the increase in susceptibility to the Delta vs pre-Delta variant was estimated to be highest in the group aged 10 to 15 years, approximately doubling (1.92-fold increase [95% CI, 1.86-fold to 1.98-fold]), whereas in the group aged 50 years or more, susceptibility to the Delta vs pre-Delta variant remained stable at an approximately 1-fold change (eg, among individuals aged 50-55 years: 0.997-fold [95% CI, 0.989-fold to 1.001-fold).

**Conclusions and Relevance:**

In this study, the Delta variant of SARS-CoV-2 was estimated to propagate more easily among children and adolescents than pre-Delta strains, even after adjusting for contact pattern and vaccination status.

## Key Points

**Question:**

Is the Delta variant (B.1.617.2) more transmissible than previous strains of SARS-CoV-2 among children and adolescents?

**Findings:**

This decision analytic model of 106 866 confirmed COVID-19 infections found that the increase in susceptibility to the Delta vs pre-Delta variant was highest in the group aged 10 to 15 years, with an increase of 1.92-fold.

**Meaning:**

This study found that even after adjusting for contact pattern and vaccination status, the Delta variant of SARS-CoV-2 was estimated to propagate more easily among children than pre-Delta strains.

## Teaser

This decision analytic model investigates susceptibility to the Delta variant of SARS-CoV-2 vs previous variants by age group.

## Introduction

A recent study found that the Delta variant (B.1.617.2) of SARS-CoV-2 had 97% (95% CI, 76%-117%) increased transmissibility compared with the previous circulating lineage.^1^ In particular, reports of increased COVID-19 infections among children and adolescents are noteworthy.^2,3^ In the United States, where the Delta variant has been predominant since late June 2021, COVID-19 infections among individuals aged 0 to 17 years increased from June to August 2021. Daily incidence rates among individuals aged 0 to 4 years, 5 to 11 years, and 12 to 17 years in June 2021 were 1.7 infections, 1.9 infections, and 2.9 infections per 100 000 individuals, respectively, and increased to 6.2 infections, 28.5 infections, and 32.7 infections per 100 000 individuals, respectively, in August 2021.^2^ A study from New South Wales, Australia, reported that in educational settings there was a 5-fold increased rate of transmission during the Delta wave (ie, until late July 2021) compared with 2020.^3^

However, whether increased incidence among the younger population was associated with the generally increased transmissibility of the Delta variant and whether individuals who were unvaccinated (ie, children and adolescents at that time) are more susceptible to contracting the Delta variant are yet to be elucidated. In South Korea, the fourth wave of COVID-19 has been driven by the Delta variant since late June 2021, with that variant accounting for 3.27% and 89.6% of total infections from June 20 to 26 and August 15 to 21, respectively.^4^ The proportion of infections among individuals aged 0 to 19 years increased from the third wave (11.01%) to the fourth wave (16.72%). Simultaneously, vaccination prioritized to older individuals was rolled out, and this accelerated after June 2021. Therefore, we investigated age-specific susceptibility associated with the Delta variant compared with the original virus, using data from the third wave (ie, before the Delta variant importation) and fourth wave (ie, driven by the Delta variant), along with vaccine coverage data in South Korea. The force of infection (λ*_i_*) experienced by age group *i* was used to estimate age-specific susceptibility in this study.^5^

There are several advantages to estimating age-specific susceptibility in South Korea. First, the Korean government has reported age-stratified COVID-19 incidence rates daily based on vigorous contact tracing.^6^ Considering the low seroprevalence of SARS-CoV-2–specific antibodies at 0.33% (ie, 4 in 1200 samples) in samples obtained from the Korea National Health and Nutrition Examination Survey from May to July 2021, the ascertainment ratio in South Korea may not be far from reality.^4^ Second, age-specific rates of COVID-19 vaccine uptake have also been reported, which enabled us to control confounding effects of vaccination on susceptibility.^6^ Third, to enhance accuracy regarding age-specific contact patterns, we applied weekly reports of school closure status in Korea from the Ministry of Education.^7^

## Methods

All data used in this decision analytic model were publicly available. The institutional review board of National Cancer Center Korea determined this study to be exempt from institutional review board assessment and waived the informed consent requirement because these were deidentified publicly available data. We performed and reported this analysis, whenever applicable, in accordance with the Consolidated Health Economic Evaluation Reporting Standards (CHEERS) reporting guideline.

### Data

Epidemiologic data used in this decision analytical model consisted of COVID-19 reports provided by the Ministry of Health and Welfare of South Korea.^6,8^ The Korean government reports COVID-19 infections diagnosed using SARS-CoV-2 polymerase chain reaction tests at 10-year age intervals. Age-structured population data were obtained from Statistics Korea.^9^

### Contact Matrix

We introduced the projected contact matrix by Prem et al^10^ for South Korea in our compartmental model. The model consists of contact patterns in different locations: home, school, work, and others. To capture the changes in contact patterns associated with social distancing measures, we considered school closure policies and decreased contact rates at work and other places using Google mobility data.^7,11^

School attendance has been capped at two-thirds of the student population during national distancing level 1, one-third during level 2 (except for high schools, which remained at two-thirds), and full restriction (or only remote learning), which occurred during level 3 throughout 2020. We reduced the number of contacts made in school in accordance with school closure policy during the study period. However, in 2021, full opening of schools was announced and implemented after the summer vacation. Therefore, we applied weekly reports of school closure status in elementary schools, middle schools, and high schools during the fourth wave (eTable 1 in the Supplement).^7^

Because of varying contact numbers at work and other places, we applied the observed reduction in the workplace visits indicator and the mean reduction of retail and recreation, grocery and pharmacy, and transit stations indicators of mobility data during the study period.^11,12^ The park indicator of mobility data was not adopted in the model because it was associated with seasonality rather than the COVID-19 outbreak and social distancing strategy.

### Statistical Analysis

#### Model Construction

**Figure 1.**
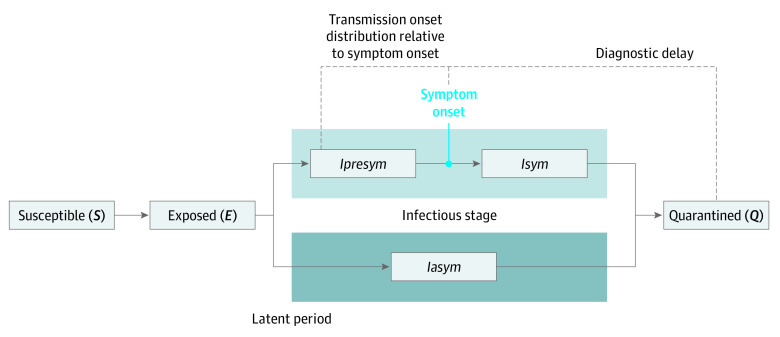
Schematic of Time Periods in Transmission of SARS-CoV-2 *Iasym* indicates infectious and asymptomatic; *Ipresym*, infectious and presymptomatic; *Isym*, infectious and symptomatic.

We built an age-structured compartment model stratified into 5-year age bands. The age-stratified (ie, at 10-year intervals) incidence of COVID-19 was made compatible with the contact matrix by dividing incidence data into 5-year age groups proportional to the demographic structure. Compartments in the model were stratified by infection states (ie, susceptible [*S*], exposed [*E*], infectious and presymptomatic [*Ipresym*], infectious and symptomatic [*Isym*], infectious and asymptomatic [*Iasym*], or quarantined [*Q*]), age band, and transition time to the next infection state (Figure 1). We assumed that people were initially susceptible (*S*) and became exposed (*E*) after an effective contact with an individual who was infectious. After a latent period, individuals who were exposed became infectious with a presymptomatic state (*Ipresym*) followed by symptomatic infection (*Isym*) or with an asymptomatic state (*Iasym*). After the infectious period, individuals entered the removed state owing to isolation, or quarantine (*Q*). In South Korea, individuals who have been diagnosed with COVID-19 are isolated immediately; thus, the confirmation date could be regarded as the date on which quarantine started. A strength of this model is that we know the diagnostic delay distribution (ie, symptom onset to *Q*), transmission onset distribution relative to symptom onset (ie, *I* given symptom onset), and latent period distribution (ie, *E* to *I*) based on the robust contact tracing study in South Korea (Table).^13,23^ We assumed that individuals who never developed symptoms (ie, *Iasym*) had the same latent period distribution as individuals who developed symptoms (ie, *Ipresym* to *Isym)* and the same infectious period distribution as the total infectious period of individuals who were symptomatic.^14^ With this backward inference method, the remaining variable to estimate was the force of infection (ie, *S* to *E*). To resolve this problem, we used a bayesian inference method. We inferred the exposure times conditional that the force of infection for each age group *i* was known and then inferred the force of infection given that the exposure times were available. We repeated these 2 steps several times until the posterior distribution converged.

**Table.** Model Parameters

Parameter	Value	Source
Incubation period	γ (μ = 4.544; k = 1/0.709)	Chun et al,^13^ 2020
Transmission onset relative to symptom onset	−4 + γ (μ = 5.266; k = 1/0.8709)	Chun et al,^13^ 2020
Latent period	Incubation period + transmission onset relative to symptom onset	Chun et al,^13^ 2020
Delay from symptom onset to diagnosis	Empirical distribution from raw data	Chun et al,^13^ 2020
Infectious period for asymptomatic infections	γ (μ = 4, k = 4/5)	Davies et al,^14^ 2020
Proportion of asymptomatic infections	16% (4% to 40%) or 52%, 50%, 45%, and 12% among individuals aged 0 to 4, 5 to 11, 12 to 17, and ≥18 y, respectively	Byambasuren et al,^15^ 2020; Dawood et al,^16^ 2022
Relative infectiousness of asymptomatic infections, %	50 (25 to 75)	Davies et al,^14^ 2020; Nakajo et al,^17^ 2021; Johansson et al,^18^ 2021
Age group, y	0 to <5, 5 to <10, 10 to <15, 15 to <20, 20 to <25, 25 to <30, 30 to <35, 35 to <40), 40 to <45, 45 to <50, 50 to <55, 55 to <60, 60 to <65, 65 to <70, 70 to <75, and ≥75	NA
Vaccine effectiveness against Delta variant infection, % (95% CI)		
BNT162b2 one dose ≥21 d	57 (50 to 63)	Lopez et al,^19^ 2021
BNT162b2 two doses >14d	80 (77 to 83)	Pouwels et al,^20^ 2021
ChAdOx1 one dose ≥21 d	46 (35 to 55)	Polinski et al,^21^ 2021
ChAdOx1 two doses >14 d	67 (62 to 71)	Bruxvoort et al,^22^ 2021
mRNA to 1273 one dose ≥21 d	75 (64 to 83)	
mRNA to 1273 two doses >14d	85 (84 to 89)	
Ad26.COV2.S >14 d	69 (67 to 71)	

Abbreviation: NA, not applicable.

According to Vynnycky and White,^24^ the force of infection λ*_i_* is written as follows in Equation 1:



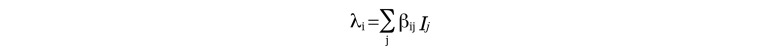



Here, β_ij_ is the rate at which individuals who are susceptible in the age group *i* and individuals who are infectious in the age group *j* come into effective contact per unit time. *I_j_* is the number of individuals who are infectious in age group *j*. We further divide *β_ij_* in Equation 2:



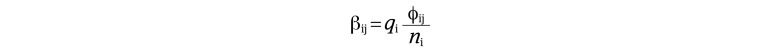



Here, *q_i_* is the probability that a contact between an individual who is susceptible in age group *i* and a person who is infectious leads to infection, ϕ_ij_ is the number of contacts an individual in age group *j* makes with those in age group *i* per unit time, and *n_i_* is the number of individuals in age group *i*. Because we knew the contact matrix for South Korea and age-stratified incidence of COVID-19 at discrete time *t*, we could infer λ_i_ (accordingly, *q_i_*) of age group *i*.^10,13^ Detailed bayesian inference methods are available in eMethods in the Supplement. All analyses were conducted using R statistical software version 3.6.3 (R Project for Statistical Computing). We provide 95% CIs using the bayesian inference method. For code and data to reproduce the analyses, see eMethods in the Supplement. Data were analyzed from September to November 2021.

#### Study Period

**Figure 2.**
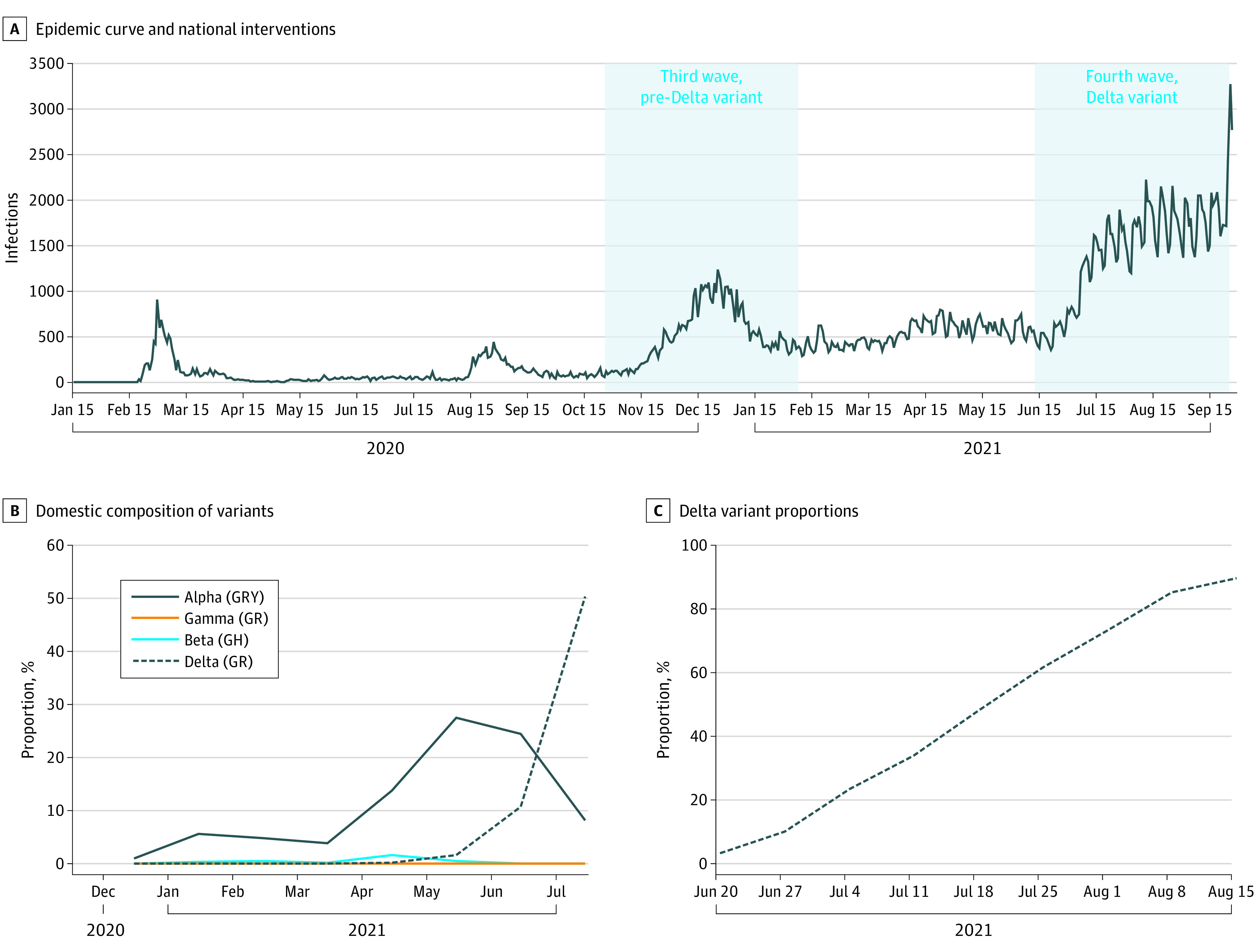
Characteristics of SARS-CoV-2 Outbreak in South Korea The social distancing level for schools was set at two-thirds density on October 12, 2020, and two-thirds density for high schools and one-third density for other schools on November 24, 2020; a stepwise opening was initiated for schools on July 12, 2021. Social distancing level was adjusted by Google mobility data for work and other sites.

We aimed to compare *q_i_* during the third COVID-19 wave, from October 15 to December 22, 2020 (when the Delta variant had not evolved and the COVID-19 vaccination campaign was not yet launched) and the fourth wave, from June 27 to August 21, 2021, in Korea (Figure 2). As vaccine uptake increased, we excluded individuals who were immunized from the susceptible population in accordance with vaccine effectiveness against the Delta variant (Table). In detail, age-specific vaccine coverage data by vaccine type and doses have been reported weekly by the Ministry of Health and Welfare of South Korea (eTable 2 and eTable 3 in the Supplement).^8^ We divided the weekly number of immunized individuals by 7 to obtain a daily number of immunized individuals for the corresponding week and removed them from the susceptible population 2 weeks after vaccination, taking into consideration the time to achieve immunity against COVID-19.

#### Sensitivity Analysis

There are uncertainties about these model parameters, including the proportion of individuals who were infected and asymptomatic, ascertainment ratio of children and adolescents, relative infectiousness of individuals who were infected and asymptomatic, and vaccine effectiveness. Therefore, we varied those values with a sensitivity analysis. A meta-analysis^15^ found that the proportion of individuals who were infected and asymptomatic ranged from 4% to 41%; hence, we varied the parameter from 4% to 40%. In addition, we attempted to vary the proportion of asymptomatic infections by age following a prospective household cohort study (ie, 52%, 50%, 45%, and 12% among individuals aged 0-4 years, 5-11 years, 12-17 years, and ≥18 years, respectively).^16^ Furthermore, given that younger individuals are more likely to be asymptomatic and thus undiagnosed, we increased infection numbers among those aged younger than 20 years to be 1.2-fold greater than those reported. The relative infectiousness of asymptomatic infections was ranged from 25% to 75%.^17,18^ For vaccine effectiveness, we adopted lower and upper bounds of the 95% CI for sensitivity analyses as reported in other studies.^19,20,21,22^

## Results

### Age Distribution of COVID-19 in South Korea

**Figure 3.**
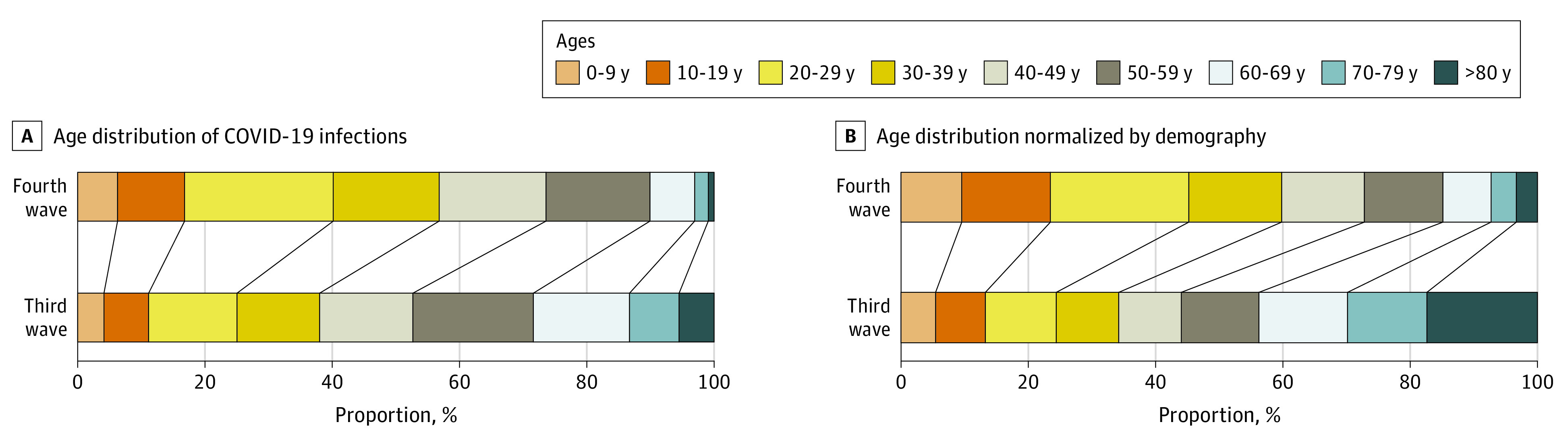
Age Distribution of COVID-19 Infections

Among 106 866 confirmed COVID-19 infections, there were 26 597 infections in the third wave and 80 269 infections in the fourth wave. The age distribution of COVID-19 infections in years is shown in Figure 3. The proportion of COVID-19 infections among those aged 19 years or less was 2928 infections (11.01%) during the third wave and 13 422 infections (16.72%) during the fourth wave, while the proportion of infections among those aged 60 years or more was 7564 infections (28.44%) during the third wave and 8156 infections (10.16%) during the fourth wave. Considering age demographics (age skewed older in South Korea^9^), the proportion of infections among individuals aged 19 years or less as normalized by age demographics was 13.28% during the third wave and 23.43% during the fourth wave.

### Age-Specific Susceptibility to COVID-19 (pre-Delta vs Delta)

**Figure 4.**
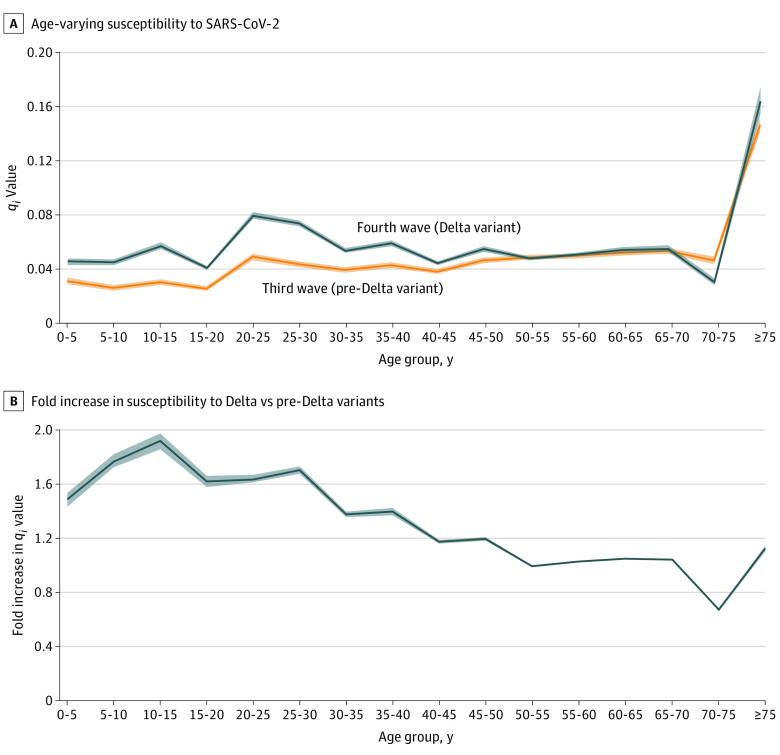
Age-Varying Susceptibility and Fold Increase in Susceptibility by Age Group Shaded areas indicate 95% CIs; *q_i_*, age-specific susceptibility.

The age-specific susceptibility (*q_i_*) to COVID-19, that is the age-stratified probability that a person who was susceptible in age group *i* acquired infection given contact with a person who was infectious, is shown in Figure 4. The third (ie, pre-Delta) wave and fourth (ie, Delta-driven) wave had similar age-associated increases, characterized by the sharp increase among those aged 75 years or more. A significant difference between the *q_i_* for the Delta variant and pre-Delta variant was found in the younger age group: After adjustment for contact pattern and vaccination status, the increase in susceptibility to the Delta variant vs the pre-Delta variant was highest in the group aged 10 to 15 years, approximately doubling (1.92-fold increase [95% CI, 1.86-fold to 1.98-fold]), whereas in those aged 50 years or more, susceptibility to the Delta vs pre-Delta remained stable at an approximately 1-fold change, except for ages 70 to less than 75 years. The fold change was 0.997 (95% CI, 0.989 to 1.001) for ages 50 to less than 55 years, 1.029 (95% CI, 1.023 to 1.040) for ages 55 to less than 60 years, 1.052 (95% CI, 1.051 to 1.052) for ages 60 to less than 65 years, 1.045 (95% CI, 1.045 to 1.048) for ages 65 to less than 70 years, 0.674 (95% CI, 0.663 to 0.682) for ages 70 to less than 75 years, and 1.126 (95% CI, 1.107 to 1.151) for ages 75 years or older.

### Sensitivity Analysis

Varying the proportion of asymptomatic infections, ascertainment ratio of children and adolescents, and relative infectiousness of asymptomatic infections, the increase in susceptibility (*q_i_*) to the Delta variant vs pre-Delta variant did not change, with the highest value in the group aged 10 to 15 years. The results were unchanged when varying vaccine effectiveness from the lower bound to the upper bound of the 95% CI (eFigures 1-9 in the Supplement).

## Discussion

The Delta variant is estimated to be more transmissible than previous strains of SARS-CoV-2, especially among children and adolescents. However, whether this is true and, if so, how much more transmissible this variant is has not been documented. COVID-19 infections among children and adolescents may be increased compared with among adults because school-aged children and adolescents have increased contact rates compared with other age groups and the current COVID-19 vaccination strategy prioritizes older people in most countries.^4,10,25,26^ To estimate the susceptibility among children and adolescents more precisely in this decision analytic model, we constructed an age-structured compartmental model (SEIQ) and included age-specific vaccine uptake rates in South Korea. With known intervals between each compartment (ie, *E* to *I* and *I* to *Q*) in South Korea, we estimated the force of infection (ie, *S* to *E*) for an individual in age group *i* using a bayesian inference method.

Even after adjusting for contact pattern and vaccination status, age-specific susceptibility among individuals aged 10 to 15 years was increased approximately 2-fold during the fourth (ie, Delta-driven) wave compared with the third wave. Indeed, children and adolescents were estimated to be more susceptible to the Delta variant compared with the original strain of SARS-CoV-2. However, this does not necessarily suggest that children and adolescents had increased risk from Delta vs the original variant. In the United States, the proportion of children and adolescents admitted to the intensive care unit for COVID-19 during the pre-Delta period (ie, March 1, 2020 to June 19, 2021) and the Delta-predominant period (ie, June 20 to July 31, 2021) did not differ among those with COVID-19–associated hospitalizations (26.5% and 23.2%, respectively).^2^ A study from Australia^3^ reported that although many children and adolescents were infected with the Delta variant, most children (98%) had asymptomatic or mild infections. In South Korea, no fatality had been observed among individuals aged 0 to 19 years from the first COVID-19 importation, on January 19, 2020, until October 4, 2021.^6^

### Limitations

There are some limitations to this study. First, the contact matrix in this study did not use our own empirical data but instead mathematically estimated data. Furthermore, although we attempted to modify this original (ie, prepandemic) contact matrix to apply social distancing measures, it may be insufficient to reflect reality. What is more, the implementation of nonpharmaceutical interventions could change the effective contact rates during the pandemic, which we could not include in this study. Second, the number of confirmed COVID-19 infections was considered the real number of infections because the South Korean case fatality ratio before vaccination rollout was close to the known infection fatality ratio of 1.15% (95% prediction interval range, 0.78%-1.79%) in high-income countries and because the latest (ie, as of July 2021) national seroprevalence of SARS-CoV-2 antibodies was 0.33%.^4,27^ However, this assumption may not be accurate. Third, the exact proportion of asymptomatic infections remained unclear. We adopted results from a meta-analysis^15^ and further conducted a sensitivity analysis with variable ranges of asymptomatic proportions. Furthermore, there may be a concern that children and adolescents usually experience asymptomatic or paucisymptomatic infections and that the ascertainment ratio for children and adolescents would be low. Nonetheless, the previously listed national seroprevalence study results (ie, 4 of 1200 samples [0.33%]) suggest that it is unlikely that we overlooked overt infections among children and adolescents. We also included an additional sensitivity analysis assuming fewer reported infections among individuals aged younger than 20 years, with results that remained consistent. Fourth, we did not account for the waning of vaccine-induced immunity in this study. However, children and adolescents (ie, those aged <18 years) had not yet been included in the national vaccination campaign against COVID-19 during the study period. Therefore, this could be assumed not to have affected the results directly. However, it may have affected them indirectly through adults with whom children and adolescents made contact.

## Conclusions

The large-scale testing and prompt epidemiological survey, as well as the recording of vaccination status in a national register in South Korea, allowed us to analyze age-stratified susceptibility to COVID-19. Generally, in this study, the Delta variant of SARS-CoV-2 was estimated to propagate more easily among children and adolescents compared with pre-Delta strains.

## Notes

Supplement. **eMethods.** Bayesian Inference Method to Estimate Age-Varying Susceptibility to SARS-CoV-2 **eTable 1.** School Attendance Ratio During Fourth Wave **eTable 2.** Vaccine Coverage Data First Half of 2021 **eTable 3.** Vaccine Coverage Data Second Half of 2021 **eFigure 1.** Result of Sensitivity Analysis: Baseline **eFigure 2.** Result of Sensitivity Analysis: 4% Asymptomatic Infections **eFigure 3.** Result of Sensitivity Analysis: 40% Asymptomatic Infections **eFigure 4.** Result of Sensitivity Analysis: Age-Varying Asymptomatic Proportions **eFigure 5.** Result of Sensitivity Analysis: Age-Varying Ascertainment Ratio **eFigure 6.** Result of Sensitivity Analysis: 25% Relative Infectiousness of Asymptomatic Infections **eFigure 7.** Result of Sensitivity Analysis: 75% Relative Infectiousness of Asymptomatic Infections **eFigure 8.** Result of Sensitivity Analysis: Vaccine Efficacy at Lower Bound of 95% CI **eFigure 9.** Result of Sensitivity Analysis: Vaccine Efficacy at Upper Bound of 95% CI

## References

[1] Campbell F, Archer B, Laurenson-Schafer H, . Increased transmissibility and global spread of SARS-CoV-2 variants of concern as at June 2021. Euro Surveill. 2021;26(24):2100509. doi:10.2807/1560-7917.ES.2021.26.24.2100509 34142653 PMC8212592

[2] Siegel DA, Reses HE, Cool AJ, ; MAPW1. Trends in COVID-19 cases, emergency department visits, and hospital admissions among children and adolescents aged 0-17 years—United States, August 2020-August 2021. MMWR Morb Mortal Wkly Rep. 2021;70(36):1249-1254. doi:10.15585/mmwr.mm7036e1 34499628 PMC8437056

[3] National Centre for Immunisation Research and Surveillance (NCIRS) Australia. COVID-19 Delta variant in schools and early childhood education and care services in NSW, Australia: 16 June to 31 July 2021. Accessed October 3, 2021. https://www.ncirs.org.au/covid-19-delta-variant-schools-and-early-childhood-education-and-care-services-nsw-australia-16

[4] Korea Disease Control and Prevention Agency. Press release: Korea COVID-19 update. Accessed October 4, 2021. https://www.kdca.go.kr/index.es?sid=a3

[5] Davies NG, Klepac P, Liu Y, Prem K, Jit M, Eggo RM; CMMID COVID-19 working group. Age-dependent effects in the transmission and control of COVID-19 epidemics. Nat Med. 2020;26(8):1205-1211. doi:10.1038/s41591-020-0962-9 32546824

[6] Ministry of Health and Welfare. Coronavirus disease-19, Republic of Korea. Accessed October 4, 2021. http://ncov.mohw.go.kr/

[7] Ministry of Education Republic of Korea. Remote learning and school opening information. Accessed October 4, 2021. https://www.moe.go.kr/boardCnts/listRenew.do?boardID=72754&m=031302&s=moe

[8] Korea Disease Control and Prevention Agency. COVID-19 vaccination. Accessed January 2, 2022. https://ncv.kdca.go.kr/eng/

[9] Korean Statistical Information Service. Population projection for Korea. Accessed January 10, 2021. https://kosis.kr/statisticsList/statisticsListIndex.do?menuId=M_01_01&vwcd=MT_ZTITLE&parmTabId=M_01_01#SelectStatsBoxDiv

[10] Prem K, Zandvoort KV, Klepac P, ; Centre for the Mathematical Modelling of Infectious Diseases COVID-19 Working Group. Projecting contact matrices in 177 geographical regions: an update and comparison with empirical data for the COVID-19 era. PLoS Comput Biol. 2021;17(7):e1009098. doi:10.1371/journal.pcbi.1009098 34310590 PMC8354454

[11] Google. COVID-19 community mobility reports. Accessed October 4, 2021. https://www.google.com/covid19/mobility/

[12] Davies NG, Barnard RC, Jarvis CI, ; Centre for Mathematical Modelling of Infectious Diseases COVID-19 Working Group; ISARIC4C investigators. Association of tiered restrictions and a second lockdown with COVID-19 deaths and hospital admissions in England: a modelling study. Lancet Infect Dis. 2021;21(4):482-492. doi:10.1016/S1473-3099(20)30984-1 33357518 PMC7758181

[13] Chun JY, Baek G, Kim Y. Transmission onset distribution of COVID-19. Int J Infect Dis. 2020;99:403-407. doi:10.1016/j.ijid.2020.07.075 32771633 PMC7409940

[14] Davies NG, Kucharski AJ, Eggo RM, Gimma A, Edmunds WJ; Centre for the Mathematical Modelling of Infectious Diseases COVID-19 working group. Effects of non-pharmaceutical interventions on COVID-19 cases, deaths, and demand for hospital services in the UK: a modelling study. Lancet Public Health. 2020;5(7):e375-e385. doi:10.1016/S2468-2667(20)30133-X 32502389 PMC7266572

[15] Byambasuren O, Cardona M, Bell K, Clark J, McLaws M-L, Glasziou P. Estimating the extent of asymptomatic COVID-19 and its potential for community transmission: systematic review and meta-analysis. J Assoc Med Microbiol Infect Dis Can. 2020;5(4):223-234. doi:10.3138/jammi-2020-0030 PMC9602871 36340059

[16] Dawood FS, Porucznik CA, Veguilla V, . Incidence Rates, household infection risk, and clinical characteristics of SARS-CoV-2 infection among children and adults in Utah and New York City, New York. JAMA Pediatr. 2022;176(1):59-67. doi:10.1001/jamapediatrics.2021.4217 34623377 PMC8501415

[17] Nakajo K, Nishiura H. Transmissibility of asymptomatic COVID-19: data from Japanese clusters. Int J Infect Dis. 2021;105:236-238. doi:10.1016/j.ijid.2021.02.065 33618004 PMC7894083

[18] Johansson MA, Quandelacy TM, Kada S, . SARS-CoV-2 transmission from people without COVID-19 symptoms. JAMA Netw Open. 2021;4(1):e2035057-e2035057. doi:10.1001/jamanetworkopen.2020.35057 33410879 PMC7791354

[19] Lopez Bernal J, Andrews N, Gower C, . Effectiveness of Covid-19 vaccines against the B.1.617.2 (Delta) variant. N Engl J Med. 2021;385(7):585-594. doi:10.1056/NEJMoa2108891 34289274 PMC8314739

[20] Pouwels KB, Pritchard E, Matthews PC, . Effect of Delta variant on viral burden and vaccine effectiveness against new SARS-CoV-2 infections in the UK. Nat Med. 2021;27(12):2127-2135. doi:10.1038/s41591-021-01548-7 34650248 PMC8674129

[21] Polinski JM, Weckstein AR, Batech M, Effectiveness of the single-dose Ad26.COV2.S COVID Vaccine. medRxiv. Preprint posted online September 16, 2021. doi:10.1101/2021.09.10.21263385

[22] Bruxvoort KJ, Sy LS, Qian L, Effectiveness of mRNA-1273 against Delta, Mu, and other emerging variants. medRxiv. Preprint posted online October 1, 2021. doi:10.1101/2021.09.29.21264199 PMC8671836 34911691

[23] Chun JY, Jeong H, Beutels P, Ohmagari N, Kim Y, Tsuzuki S. COVID-19 vaccine prioritisation in Japan and South Korea. medRxiv. Preprint posted online April 20, 2021. doi:10.1101/2021.04.16.21255649

[24] Vynnycky E, White R. An Introduction to Infectious Disease Modelling. Oxford University Press; 2010.

[25] Dooling K, Marin M, Wallace M, . The Advisory Committee on Immunization Practices’ updated interim recommendation for allocation of COVID-19 Vaccine—United States, December 2020. MMWR Morb Mortal Wkly Rep. 2021;69(5152):1657-1660. doi:10.15585/mmwr.mm695152e2 33382671 PMC9191902

[26] European Centre for Disease Prevention and Control. Overview of COVID-19 vaccination strategies and vaccine deployment plans in the EU/EEA and the UK—2 December 2020. Accessed February 15, 2022. https://www.ecdc.europa.eu/sites/default/files/documents/Overview-of-EU_EEA-UK-vaccination-deployment-plans.pdf

[27] Brazeau NF, Verity R, Jenks S, Report 34—COVID-19 infection fatality ratio estimates from seroprevalence. Accessed February 9, 2022. https://www.imperial.ac.uk/mrc-global-infectious-disease-analysis/covid-19/report-34-ifr/

