# Supplementary material for: Age-Varying Susceptibility to the Delta Variant (B.1.617.2) of SARS-CoV-2
Source: JAMA Netw Open. 2022 Mar 18;5(3):e223064. doi: 10.1001/jamanetworkopen.2022.3064 (PMC8933737; doi:10.1001/jamanetworkopen.2022.3064)
Supplement: Supplement. — eMethods. Bayesian Inference Method to Estimate Age-Varying Susceptibility to SARS-CoV-2 eTable 1. School Attendance Ratio During Fourth Wave eTable 2. Vaccine Coverage Data First Half of 2021 eTable 3. Vaccine Coverage Data Second Half of 2021 eFigure 1. Result of Sensitivity Analysis: Baseline eFigure 2. Result of Sensitivity Analysis: 4% Asymptomatic Infections eFigure 3. Result of Sensitivity Analysis: 40% Asymptomatic Infections eFigure 4. Result of Sensitivity Analysis: Age-Varying Asymptomatic Proportions eFigure 5. Result of Sensitivity Analysis: Age-Varying Ascertainment Ratio eFigure 6. Result of Sensitivity Analysis: 25% Relative Infectiousness of Asymptomatic Infections eFigure 7. Result of Sensitivity Analysis: 75% Relative Infectiousness of Asymptomatic Infections eFigure 8. Result of Sensitivity Analysis: Vaccine Efficacy at Lower Bound of 95% CI eFigure 9. Result of Sensitivity Analysis: Vaccine Efficacy at Upper Bound of 95% CI [file jamanetwopen-e223064-s001.pdf]

Supplemental Online Content

Chun JY, Jeong H, Kim Y. Age-varying susceptibility to the Delta variant (B.1.617.2) of SARS-CoV-2. *JAMA Netw Open*. 2022;5(3):e223064. doi:10.1001/jamanetworkopen.2022.3064

eMethods. Bayesian Inference Method to Estimate Age-Varying Susceptibility to SARS-CoV-2

eTable 1. School Attendance Ratio During Fourth Wave

eTable 2. Vaccine Coverage Data First Half of 2021

eTable 3. Vaccine Coverage Data Second Half of 2021

eFigure 1. Result of Sensitivity Analysis: Baseline

eFigure 2. Result of Sensitivity Analysis: 4% Asymptomatic Infections

eFigure 3. Result of Sensitivity Analysis: 40% Asymptomatic Infections

eFigure 4. Result of Sensitivity Analysis: Age-Varying Asymptomatic Proportions

eFigure 5. Result of Sensitivity Analysis: Age-Varying Ascertainment Ratio

eFigure 6. Result of Sensitivity Analysis: 25% Relative Infectiousness of Asymptomatic Infections

eFigure 7. Result of Sensitivity Analysis: 75% Relative Infectiousness of Asymptomatic Infections

eFigure 8. Result of Sensitivity Analysis: Vaccine Efficacy at Lower Bound of 95% CI

eFigure 9. Result of Sensitivity Analysis: Vaccine Efficacy at Upper Bound of 95% CI

This supplemental material has been provided by the authors to give readers additional information about their work.

© 2022 Chun JY et al. *JAMA Network Open.*

eMethods. Bayesian Inference Method to Estimate Age-Varying Susceptibility to SARS-CoV-2

Bayesian Inference

A. Overview

In a Susceptible-Exposed-Infectious-Recovered (SEIR) model, the force of infection λ, the rate at which susceptible individuals are infected (i.e exposed), is a crucial factor. The age-specific force of infection λ_i in age group i at discrete time t could be written as:

$$\lambda_i = \mu(t|q_i) = \frac{q_i \sum_{j=1}^{A} \phi_{ij}(t) \left(I_{presym}^{j}(t-1) + I_{sym}^{j}(t-1) + 0.5 I_{asym}^{j}(t-1) \right)}{n_i} \quad (S1)$$

where q_i is the probability that a contact between a susceptible in age group i and infectious person leads to infection, $\phi(t) = \phi_{ij}(t)$ means contact matrix at discrete time t ($\phi_{ij}(t)$ is the number of contacts an individual of age group j makes with those of age group i per unit time at discrete time t), n_i is the number of individuals in age group i. I_{presym} is the number of individuals who become infectious before the symptom onset; I_{sym} is the number of individuals who are both symptomatic and infectious; I_{asym} is the number of individuals who are infectious but never developed any symptoms. We suppose that the relative infectiousness of the I_{asym} is half of I_{presym} or I_{sym}. Here i is in age group $\underline{A} = \{1, 2, \cdots, A\}$.

We try to estimate q_i during the third and fourth waves in South Korea and denote it as θ_i hereafter. If we could observe the number of exposed individuals at time t for age group i and the number of total infectious individuals, the likelihood of the parameters could be easily derived. However what we could observe is only the number of diagnosed (i.e quarantined) individuals for age group $i \in \underline{A}$ at time t. In addition, asymptomatic infection which is the notable feature of COVID-19 should also be reflected in the model. To resolve these difficulties, we use a Bayesian approach. In particular, we develop an efficient MCMC (Markov Chain Monte Carlo) algorithm in which the exposed date, symptom onset date and transmission onset date for all quarantined individuals are imputed with an assumption that there are 16% asymptomatic individuals. We explain the details of our Bayesian method in the following three subsections.

B. Data, Model and Posterior

The data we used in the analysis is daily numbers of quarantined individuals for each age group from 15 October to 22 December 2020 (3rd wave) and from 27 June to 21 August 2021 (4th wave).

To estimate θ_i, we are going to impute the exposed dates, symptom onset dates and transmission onset dates of all quarantined individuals conditional on given quarantined dates. For this purpose, we need a probability model which relates the exposed dates, symptom onset dates and transmission onset dates to the quarantined dates.

**Symptomatic cases**		**Asymptomatic cases**	
**Symbol**	**Definition**	**Symbol**	**Definition**
E	Exposed date of an individual	***E***	Exposed date of an individual
Y	Incubation period	***L***	Latent period
I	Transmission onset time relative to the symptom onset	***C***	Infectious period suspended by quarantine
D	Diagnostic delay (i.e Quarantined) from the symptom onset	***R***	Infectious period

For each symptomatic individual, the quarantined date is sum of the exposed date(E), the incubation period(Y) and the period for symptom onset to diagnostic delay(D). In addition, these individuals start infecting other susceptibles from the transmission onset date ($E + Y + I$)

For each asymptomatic individual, the quarantined date is sum of exposed date (E), latent period(L) and period for transmission onset to quarantined date(C). We assume that the latent period distribution of asymptomatic individuals is same as that of symptomatic individuals. Also the quarantined time distribution of $C(f_C)$ of asymptomatic individuals is set to be the exponential distribution with mean 1/1.7, which satisfies $P(C > R) = 0.01$ where $E + L + R$ is defined as the recovered date.

Finally, the quarantined date T is defined as

$$T = (E + Y + D)\boldsymbol{I}(\Delta = 0) + (E + L + C)\boldsymbol{I}(\Delta = 1) \quad (S2)$$

where Δ is equal to 0 when the individual is symptomatic and 1 when asymptomatic.We set $P(\Delta = 1) = 0.16$.

For individual k, let $W_k = (E_k, Y_k, I_k, L_k, C_k, D_k)$ and $V_k = (Y_k, I_k, L_k, C_k, D_k)$. Let $\underline{D}$ be the observed data which consist of the daily numbers of quarantined individuals. Our strategy to estimate θ_i is to generate W_k and θ iteratively from their conditional posterior distributions $P(\theta_i|W, \underline{D})$ and $P(W_k|W_{(-k)}, \theta, \underline{D})$ respectively, where $W = \{W_k\}$ and $W_{(-k)}$ denotes W except W_k .

We could describe as,

$$P\big(W_k\big|W_{(-k)}, \theta_{i_k}, \underline{D}\big) = P\big(W_k\big|W_{(-k)}, \theta_{i_k}, D\big)\boldsymbol{I}(\Delta_k = 0) + P\big(W_k\big|W_{(-k)}, \theta_{i_k}, D\big)\boldsymbol{I}(\Delta_k = 1) \quad (S3)$$

$$= P(E_k|V_{(1:N)}, \theta_{i_k}, D)(f_Y(Y_k)f_I(I_k)f_D(D_k)\boldsymbol{I}(\Delta_k = 0) + f_L(L_k)f_C(C_k)\boldsymbol{I}(\Delta_k = 1)) \quad (S4)$$

C. Generating θ and W from their conditional posterior distributions

C.1. Generating $\boldsymbol{\theta}$

For the prior distribution of θ_i we use a diffuse gamma distribution $Gamma(0.001, 0.001)$ for all i. Then

$$p(\theta_i|W^i_{(1:n_i)}, \underline{D}) \propto p(\theta_i)p(W^i_{(1:n_i)}, \underline{D}|\theta_i), \ i \in \underline{A} \quad (S5)$$

when $W^i = \{W_k \mid The\ age\ group\ of\ k\ is\ i\}$, n_i is the population for age group i.

In turn, $P(W^i_1, \cdots, W^i_{n_i}, \underline{D}|\theta_i)$ can be expressed as

$$P\big(W^i_1, \cdots, W^i_{n_i}, \underline{D}\big|\theta_i\big) = P\big(E^i_1, \cdots, E^i_{n_i}\big|V_{(1:N)}, \theta_i\big)P\big(V_{(1:N)}\big) = \prod_{k=1}^{n_i} P\big(E^i_k\big|\, V_{(1:N)}, \theta_i\big)P\big(V_{(1:N)}\big) \quad (S6)$$

$$P(E^i_k|\, V_{(1:N)}, \theta_i) = P(E^i_k|\, I^j_{total}(t), t < E^i_k, \theta_i) = p(E^i_k \mid \theta_i)\textstyle\prod_{t=1}^{E^i_k - 1}(1 - p(t|\theta_i)) \quad (S7)$$

Since $p(E^i_k|V_{(1:N)}, \theta_i)$ is the probability of an individual k to be infected at discrete time E^i_k (implying the individual k has not been infected before).

$$P\big(V_{(1:N)}\big) = \prod_{\Delta_k = 0} 0.84\, f_Y(Y_k)f_I(I_k)f_D(D_k) \prod_{\Delta_k = 1} 0.16\, f_L(L_k)f_C(C_k) \quad (S8)$$

where $N = \sum_i n_i$ (Total population size),$V_k = (Y_k, I_k, L_k, C_k, D_k)$ and i_k is the age group of individual k. By applying the following approximation,

$$P(E^i_k|V_{(1:N)}, \theta_{i_k}) = P(E^i_k|\, I^j_{total}(t), t < E^i_k, \theta_{i_k}) = p(E^i_k \mid \theta_{i_k}) \prod_{t=1}^{E^i_k - 1}(1 - p(t|\theta_{i_k}))$$

$$\approx p(E^i_k \mid \theta_{i_k})e^{-\sum_{t=1}^{E^i_k - 1} p(t|\theta_{i_k})} \quad (S9)$$

© 2022 Chun JY et al. *JAMA Network Open.*

we have

$$\theta_i \mid W, D$$
$$\sim Gamma\left(0.001 + |\mathcal{E}_i^*|, 0.001 + \sum_{k \in \mathcal{E}_i^*} \frac{\sum_{t_1}^{E_k} \phi_{ij}(t) I_{total}^j(t-1)}{n_i} + \sum_{k \in \{s: E_s > t_2 \ or \ E_s = NA\}} \frac{\sum_{t_1}^{t_2} \phi_{ij}(t) I_{total}^j(t-1)}{n_i}\right) \quad (S10)$$

where $\mathcal{E}_i^*$ is set of individuals in the age group i exposed during the 3rd wave $[t_1, t_2]$ (or 4th wave).

C.2 Generating W

We generate W by generating W_k from $P(W_k|W_{(-k)}, \theta, \underline{D})$ iteratively, and generate W_k through the Metropolis-Hasting (MH) algorithm. To sample from the posterior $P(W_k|W_{(-k)}, \theta, \underline{D})$ by the MH algorithm, we use the following proposal distribution:

$$Q(E_k, V_k) = Q(E_k|V_k)Q(V_k) \quad (S11)$$

$$Q(V_k) = p(V_k|\underline{D}) \quad (S12)$$

$$Q(E_k|V_k) = \delta(T_k - Y_k - D_k)\boldsymbol{I}(\Delta_k = 0) + \delta(T_k - L_k - C_k)\boldsymbol{I}(\Delta_k = 1) \quad (S13)$$

Putting the above together, the sampling procedure of W_k is summarized in Alogorithm S1.

Alogorithm S1. Bayesian Inference

Input : $W_k^{(0)}$ for $k = 1, 2, \cdots, N$

1. Sample $\theta^{(0)} = (\theta_i^{(0)})$ from prior.
2. **for** $m = 1{:}M$ (number of iteration) **do** ► **Gibbs sampling**
3. **for** $k = 1{:}N$ do ► **MCMC**
4. Sample $W_k^{(m)}$ from $Q(E_k, V_k)$
5. $\alpha \leftarrow \frac{P(E_k^{(m)}, E_{(-k)}^{(m-1)} | V_k^{(m)}, V_{(-k)}^{(m-1)}, \theta^{(m)}, \underline{D})}{P(E_k^{(m-1)}, E_{(-k)}^{(m-1)} | V_k^{(m-1)}, V_{(-k)}^{(m-1)}, \theta^{(m)}, \underline{D})}$ ► **Acceptance ratio**
6. $W_k^{(m)} \leftarrow \begin{cases} W_k^{(m)} & \alpha \geq 1 \\ W_k^{(m)} & with\,prob\ \alpha \\ W_k^{(m-1)} & else \end{cases}$
7. Sample $(\theta_i^{(m)})$ for $i = 1, 2, \cdots, A$ from $p(\theta_i|W^{(m)})$

The acceptance ratio α can be obtained as follows, where $W_k^{(m)}$ and $W_k^{(m-1)}$ are denoted by $W_k^{(new)}$ and $W_k^{(old)}$, respectively.

For I_{sym}, let $m_k^I = (T_k - D_k^{(new)} + I_k^{(new)}) \wedge (T_k - D_k^{(old)} + I_k^{(old)})$, $M_k^I = (T_k - D_k^{(new)} + I_k^{(new)}) \vee (T_k - D_k^{(old)} + I_k^{(old)})$.

For I_{asym}, let $m_k^I = (T_k - C_k^{(new)}) \wedge (T_k - C_k^{(old)})$, $M_k^I = (T_k - C_k^{(new)}) \vee (T_k - C_k^{(old)})$. Here $T - D + I$ and $T - C$ are transmission onset date and $a \wedge b = min\{a, b\}, \vee \wedge b = man\{a, b\}$.

© 2022 Chun JY et al. *JAMA Network Open.*

$$\widetilde{p_i}(t) = \begin{cases} p_i(t) & if\ t \notin [m_k^I, M_k^I] \cdots (*) \\ \dfrac{\phi_{ii_k}(I_{total}^{i_k}(t-1)-1) + \sum_{j \neq i_k} \phi_{ij} I_{total}^{j}(t-1)}{\phi_{ii_k}(I_{total}^{i_k}(t-1)) + \sum_{j \neq i_k} \phi_{ij} I_{total}^{j}(t-1)} p_i(t) & if\ not\ (*), T_k - D_k^{(new)} + I_k^{(new)} < T_k - D_k^{(old)} + I_k^{(old)} \\ \dfrac{\phi_{ii_k}(I_{total}^{i_k}(t-1)+1) + \sum_{j \neq i_k} \phi_{ij} I_{total}^{j}(t-1)}{\phi_{ii_k}(I_{total}^{i_k}(t-1)) + \sum_{j \neq i_k} \phi_{ij} I_{total}^{j}(t-1)} p_i(t) & if\ not\ (*), T_k - D_k^{(new)} + I_k^{(new)} > T_k - D_k^{(old)} + I_k^{(old)} \end{cases} \quad (S14)$$

when $A = \{ \tilde{k} : m_k^I < E_{\tilde{k}} \leq M_k^I \}$, $B = \{ \tilde{k} : E_{\tilde{k}} > M_k^I \}$

$$\alpha = \begin{cases} \dfrac{p_{i_k}(E_k^{(new)})}{p_{i_k}(E_k^{(old)})} e^{-\sum_{E_k^{(old)}}^{E_k^{(new)}-1} p_{i_k}(t)} \left(\prod_{\tilde{k} \in A} \dfrac{\widetilde{p_{i_{\tilde{k}}}}(E_{\tilde{k}})}{p_{i_{\tilde{k}}}(E_k)} \left(e^{\sum_{t=m_k^I}^{E_{\tilde{k}}-1} -\widetilde{p_{i_{\tilde{k}}}}(t) + p_{i_{\tilde{k}}}(t)} \right) \right) \prod_{\tilde{k} \in B} \left(e^{\sum_{t=m_k^I}^{M_k^I} -\widetilde{p_{i_{\tilde{k}}}}(t) + p_{i_{\tilde{k}}}(t)} \right) & E_k^{(new)} > E_k^{(old)} \\ \left(\prod_{\tilde{k} \in A} \dfrac{\widetilde{p_{i_{\tilde{k}}}}(E_{\tilde{k}})}{p_{i_{\tilde{k}}}(E_k)} \left(e^{\sum_{t=m_k^I}^{E_{\tilde{k}}-1} -\widetilde{p_{i_{\tilde{k}}}}(t) + p_{i_{\tilde{k}}}(t)} \right) \right) \prod_{\tilde{k} \in B} \left(e^{\sum_{t=m_k^I}^{M_k^I} -\widetilde{p_{i_{\tilde{k}}}}(t) + p_{i_{\tilde{k}}}(t)} \right) & E_k^{(new)} = E_k^{(old)} \\ \dfrac{p_{i_k}(E_k^{(old)})}{p_{i_k}(E_k^{(new)})} e^{-\sum_{E_k^{(new)}}^{E_k^{(old)}-1} p_{i_k}(t)} \left(\prod_{\tilde{k} \in A} \dfrac{\widetilde{p_{i_{\tilde{k}}}}(E_{\tilde{k}})}{p_{i_{\tilde{k}}}(E_k)} \left(e^{\sum_{t=m_k^I}^{E_{\tilde{k}}-1} -\widetilde{p_{i_{\tilde{k}}}}(t) + p_{i_{\tilde{k}}}(t)} \right) \right) \prod_{\tilde{k} \in B} \left(e^{\sum_{t=m_k^I}^{M_k^I} -\widetilde{p_{i_{\tilde{k}}}}(t) + p_{i_{\tilde{k}}}(t)} \right) & E_k^{(new)} < E_k^{(old)} \end{cases} \quad (S15)$$

D. Reproducibility

Code and data to reproduce the analyses are available at https://github.com/Hwichang/Age-varying-susceptibility-to-the-Delta-variant-of-SARS-CoV-2.

© 2022 Chun JY et al. *JAMA Network Open.*

eTable 1. School Attendance Ratio During Fourth Wave

Attendance ratio (%)	2021-09-29	2021-09-28	2021-09-23	2021-09-15	2021-09-13	2021-09-09	2021-09-06	2021-09-01	2021-08-31
Kindergarten	91.6	90.9	89.8	93.2	91.3	92.3	90.3	88.9	82.3
Primary school	79.7	78.7	75.6	79.3	77.9	77.7	76.4	55.7	48.6
Middle school	80.5	80.3	76.5	79.9	79.7	76.9	74	45	56.2
High school	81.4	81.7	72.9	81	80.9	80.9	81	73.5	74.2

Attendance ratio (%)	2021-08-26	2021-08-24	2021-08-19	2021-08-13	2021-08-11	2021-08-09	2021-08-04	2021-08-02	2021-07-28
Kindergarten	74.4	66.5	55.7	27.5	20.4	15.2	1.9	1.6	11.7
Primary school	36.7	20.6	4.1	0.4	0.2	0.1	0	0	0.7
Middle school	55.7	50.7	27.7	2.1	0.9	0.6	0.2	0.1	0
High school	73.1	70.1	55.9	6.5	5.2	4.2	1.1	0.9	0.4

Attendance ratio (%)	2021-07-26	2021-07-21	2021-07-16	2021-07-14	2021-07-12	2021-07-07	2021-06-30	2021-06-23	2021-06-16
Kindergarten	20.2	42.8	45.8	56.7	78	94.3	94.3	95.2	94.4
Primary school	3.2	24.5	31.5	46.9	58.6	80.2	79.9	80.2	79.3
Middle school	0.4	4.2	10.3	45.3	50.5	77.6	76.6	76.3	76
High school	0.8	3.4	10	47.3	57.6	79.4	85.6	76.2	75.7

© 2022 Chun JY et al. *JAMA Network Open.*

eTable 2. Vaccine Coverage Data First Half of 2021

Age	Type		2021-05-29	2021-06-05	2021-06-12	2021-06-19
18~29	AZ	1st	135,692	135,784	135,839	135,901
		2nd	85,495	109,495	119,623	126,856
	P	1st	27,641	29,436	178,863	416,390
		2nd	25,208	25,475	25,895	27,395
	JJ	1st				7
30~49	AZ	1st	847,219	1,025,861	1,246,444	1,408,453
		2nd	189,256	231,801	250,360	263,501
	P	1st	48,627	49,791	51,888	54,848
		2nd	45,135	45,627	46,420	48,307
	JJ	1st			528,740	993,216
50~74	AZ	1st	2,085,973	3,585,248	6,383,358	8,608,663
		2nd	224,463	258,346	297,104	335,204
	P	1st	61,363	63,194	65,844	67,835
		2nd	55,806	56,547	57,570	61,124
	JJ	1st			38,053	121,808
≥75	AZ	1st	199,117	201,748	208,756	219,906
		2nd	4,926	10,446	44,541	99,090
	P	1st	1,993,383	2,504,010	2,964,448	2,985,116
		2nd	1,513,004	1,541,859	1,583,769	1,969,791
	JJ	1st			54	312

AZ; ChAdOx1 nCoV-19 (AZD1222, Oxford–AstraZeneca), P; BNT162b2 (tozinameran, Pfizer–BioNTech), JJ; Ad26.COV2.S (Johnson & Johnson), M; mRNA-1273 (elasomeran, Moderna)

Reference: https://ncv.kdca.go.kr/

© 2022 Chun JY et al. *JAMA Network Open.*

eTable 3. Vaccine Coverage Data Second Half of 2021

Age	Type		2021-06-28	2021-07-05	2021-07-12	2021-07-19	2021-07-26	2021-08-02	2021-08-09	2021-08-16	2021-08-23	2021-08-30	2021-09-06	2021-09-13	2021-09-20
18~29	AZ	1st	135,946	135,960	135,967	136,081	136,388	137,332	137,784	138,118	138,312	138,441	138,525	138,561	138,561
		2nd	131,100	131,693	131,701	131,729	131,758	131,766	131,864	132,141	133,002	133,431	133,800	134,001	134,215
		2nd P*			70	433	828	1,102	1,251	1,430	1,628	1,811	1,942	2,031	2,315
	P	1st	623,290	626,570	695,296	759,478	1,208,724	1,502,771	1,715,833	1,964,306	2,185,802	2,697,092	3,142,071	4,003,302	4,618,031
		2nd	29,047	178,201	405,053	608,844	622,353	688,277	750,851	1,190,221	1,255,330	1,274,463	1,382,183	1,655,660	1,938,605
	JJ	1st	10	17	18	18	18	18	18	18	19	22	43	66	3,141
	M	1st	15,622	34,364	54,754	55,476	55,504	119,052	125,330	132,457	194,311	200,757	216,794	317,565	816,927
		2nd				238	13,408	33,018	54,038	55,041	55,149	56,043	65,832	119,032	141,432
30~39	AZ	1st	533,056	533,620	533,673	533,904	534,502	535,751	536,330	537,524	555,572	579,203	589,942	592,690	592,690
		2nd	128,494	136,891	136,891	136,918	137,087	137,177	137,416	137,954	139,285	140,822	142,421	142,892	143,403
		2nd P*			21,590	108,118	170,524	187,106	190,264	217,370	259,206	317,043	382,761	387,079	388,308
	P	1st	28,184	29,578	37,728	134,829	221,883	454,070	639,936	768,405	941,218	1,240,986	1,518,671	2,214,956	2,687,480
		2nd	24,546	25,257	26,102	27,000	27,851	35,236	126,569	212,461	222,234	234,213	375,123	550,202	750,133
	JJ	1st	802,334	802,874	802,951	802,956	802,959	802,983	802,983	802,987	802,987	824,599	860,571	887,563	910,527
	M	1st	1,270	3,156	4,887	5,030	5,058	43,053	46,592	51,287	99,452	104,850	121,918	217,364	593,717
		2nd					1,379	2,978	4,807	4,992	5,045	5,327	10,502	43,163	56,991
40~49	AZ	1st	880,407	881,151	881,269	881,650	882,513	883,871	884,663	887,289	927,882	985,751	1,009,579	1,014,709	1,014,709
		2nd	144,437	155,994	155,994	156,074	156,270	156,390	156,777	157,651	159,449	162,330	164,165	164,805	165,532
		2nd P*			43,974	180,789	252,570	270,534	277,512	347,791	482,002	627,500	710,976	715,286	717,390
	P	1st	29,595	31,515	40,017	218,287	335,390	682,788	936,781	1,103,408	1,353,604	1,752,595	2,121,406	3,218,448	3,898,027

© 2022 Chun JY et al. *JAMA Network Open.*

		2nd	24,958	25,940	26,932	27,931	28,893	36,630	207,346	324,335	337,429	351,209	572,692	802,695	1,084,344
	JJ	1st	196,521	197,434	197,528	197,534	197,535	197,544	197,547	197,547	197,549	218,975	251,759	276,082	300,291
	M	1st	349	960	1,581	1,643	1,648	54,227	59,161	65,864	145,300	154,611	181,244	336,632	913,232
		2nd					407	628	1,554	1,635	1,657	2,011	8,740	54,872	75,544
50~59	AZ	1st	944,250	945,353	945,945	946,353	946,766	947,206	951,653	978,718	990,173	1,006,396	1,018,618	1,022,414	1,022,727
		2nd	196,587	210,960	226,613	246,274	258,629	264,899	269,768	354,699	494,719	631,813	675,889	678,266	680,452
		2nd P*			76,942	201,471	253,240	261,198	261,450	261,717	262,016	262,493	262,710	262,800	262,891
	P	1st	39,484	41,250	48,076	168,912	254,824	1,084,096	1,752,790	2,351,468	3,675,505	4,899,921	4,924,355	4,946,820	4,966,638
		2nd	34,364	35,800	36,986	38,208	39,339	45,554	162,079	248,684	262,482	275,269	489,143	1,182,045	2,186,483
	JJ	1st	59,107	60,318	60,379	60,381	60,386	60,393	60,393	60,395	60,396	65,430	80,621	93,245	112,754
	M	1st	207	567	889	914	917	587,082	615,672	640,931	1,783,116	1,814,913	1,820,085	1,832,604	1,849,755
		2nd					249	249	671	894	923	2,255	31,553	579,658	673,075
60~69	AZ	1st	5,855,390	5,856,326	5,856,977	5,857,335	5,857,658	5,858,378	5,879,076	6,034,900	6,113,664	6,168,359	6,187,828	6,189,573	6,190,092
		2nd	139,603	150,643	161,836	180,526	192,121	197,899	204,198	704,762	1,554,773	3,816,163	5,598,836	5,629,739	5,637,075
		2nd P*			94,748	150,472	173,502	175,007	175,109	175,343	175,783	177,223	178,838	179,427	179,646
	P	1st	21,875	22,013	114,779	171,932	180,756	239,378	281,231	305,706	318,297	332,088	342,198	352,392	361,642
		2nd	20,699	21,393	21,583	21,710	21,803	110,653	166,376	176,800	177,861	179,425	193,973	245,265	293,719
	JJ	1st	60,016	60,206	60,218	60,222	60,227	60,227	60,231	60,231	60,234	61,377	65,402	68,803	76,029
	M	1st	16	61	116	125	125	44,482	46,354	47,643	58,923	59,737	61,453	66,232	72,810
		2nd					24	24	115	124	126	284	3,046	43,625	47,188
70~79	AZ	1st	1,855,808	1,856,431	1,856,784	1,856,963	1,857,142	1,857,309	1,861,424	1,891,830	1,907,389	1,918,565	1,922,785	1,923,363	1,923,581
		2nd	47,791	53,387	57,674	62,783	65,472	66,799	69,745	646,188	1,180,954	1,494,359	1,800,278	1,806,720	1,808,636

© 2022 Chun JY et al. *JAMA Network Open.*

		2nd P*			14,585	24,000	29,143	29,320	29,336	29,403	29,637	30,040	30,430	30,555	30,627
	P	1st	1,424,636	1,432,143	1,453,719	1,469,104	1,473,470	1,487,688	1,502,379	1,511,870	1,518,376	1,524,082	1,528,847	1,532,960	1,536,611
		2nd	1,115,520	1,392,073	1,405,112	1,414,527	1,422,426	1,443,725	1,458,942	1,463,773	1,464,458	1,465,530	1,468,857	1,482,547	1,500,325
	JJ	1st	8,216	8,270	8,272	8,274	8,277	8,276	8,277	8,278	8,278	8,461	9,060	9,616	10,483
	M	1st	2	9	17	17	17	9,347	9,863	10,224	12,931	13,091	13,357	14,235	15,405
		2nd					2	2	17	17	19	57	548	9,123	9,930
≥80	AZ	1st	185,863	187,493	188,398	188,827	189,188	189,447	191,547	198,708	204,109	208,934	211,177	212,076	212,355
		2nd	114,222	130,211	140,851	146,420	148,891	150,096	152,194	157,234	161,096	166,697	172,254	174,991	176,234
		2nd P*			70	188	295	301	301	301	301	303	304	305	305
	P	1st	1,588,575	1,599,254	1,606,378	1,616,518	1,620,928	1,625,155	1,631,672	1,636,340	1,641,364	1,645,645	1,650,333	1,653,144	1,655,979
		2nd	1,365,292	1,541,968	1,556,566	1,569,525	1,580,402	1,588,014	1,598,226	1,602,899	1,603,575	1,604,659	1,606,464	1,610,501	1,617,878
	JJ	1st	273	320	320	320	320	321	321	321	321	557	1,134	1,486	2,159
	M	1st		1	1	1	1	131	149	200	328	354	393	521	770
		2nd							1	1	1	1	4	125	169

* Since mid-July 2021, a mixed combination of ChAdOx1 nCoV-19 (AZD1222, Oxford–AstraZeneca) first and BNT162b2 (tozinameran, Pfizer–BioNTech) second was used.

© 2022 Chun JY et al. *JAMA Network Open.*

eFigure 1. Result of Sensitivity Analysis: Baseline

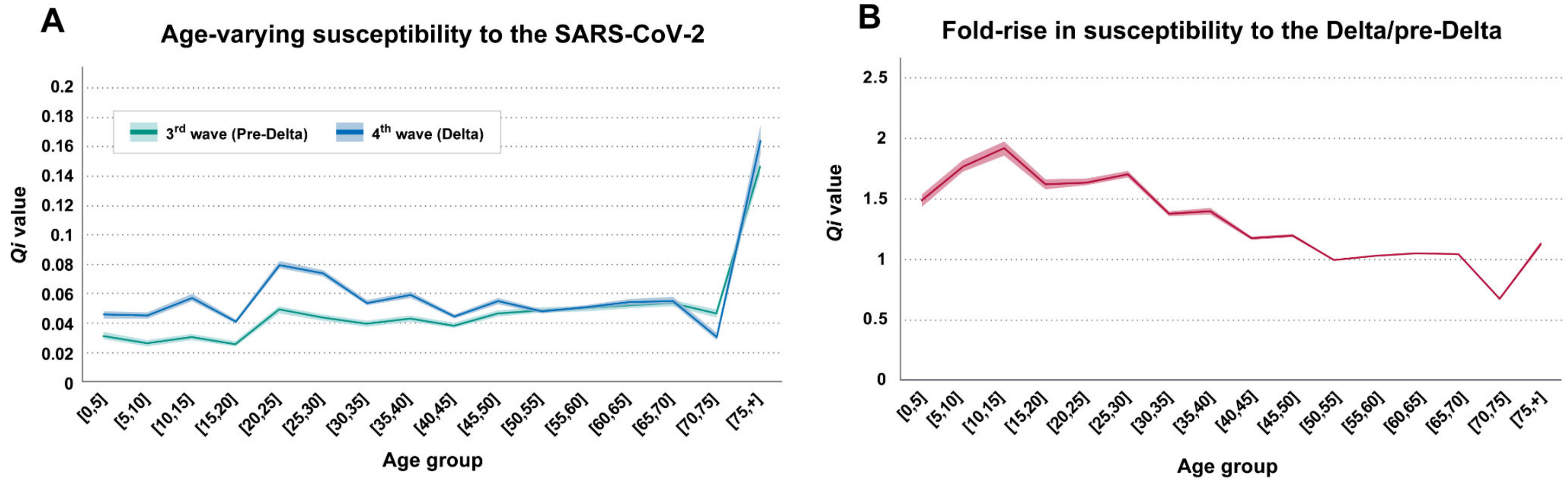

	3rd wave (Pre-Delta)			*4th wave (Delta)*			*Fold-rise*		
	2.50%	50%	97.50%	2.50%	50%	97.50%	2.50%	50%	97.50%
[0,5)	0.02829	0.030807	0.033569	0.043465	0.045887	0.048048	1.536416	1.489504	1.431315
[5,10)	0.023685	0.025646	0.027586	0.043276	0.045437	0.047555	1.82717	1.771664	1.723862
[10,15)	0.027847	0.02998	0.032231	0.055012	0.057505	0.059875	1.975501	1.918102	1.857688
[15,20)	0.023795	0.02538	0.027121	0.039571	0.041149	0.042844	1.66301	1.621304	1.579741
[20,25)	0.046352	0.048611	0.051073	0.077432	0.079681	0.082327	1.670517	1.639143	1.611961
[25,30)	0.04139	0.043289	0.045337	0.07181	0.073878	0.076013	1.734952	1.706649	1.676628

© 2022 Chun JY et al. *JAMA Network Open.*

[30,35)	0.037224	0.039097	0.040973	0.051992	0.05386	0.055663	1.396753	1.377602	1.358515
[35,40)	0.040271	0.04238	0.044537	0.057469	0.05928	0.061105	1.427043	1.398781	1.372018
[40,45)	0.036144	0.037729	0.039539	0.04292	0.04438	0.045879	1.187476	1.176285	1.160361
[45,50)	0.043949	0.045958	0.047976	0.053215	0.055067	0.05688	1.21083	1.198201	1.185606
[50,55)	0.04641	0.048158	0.050051	0.046457	0.048016	0.049475	1.001017	0.997043	0.9885
[55,60)	0.047279	0.049315	0.051147	0.049173	0.050767	0.052315	1.040061	1.029443	1.022836
[60,65)	0.049461	0.051628	0.053608	0.052036	0.054286	0.056405	1.052075	1.051481	1.052176
[65,70)	0.050556	0.053013	0.055442	0.052981	0.055411	0.058047	1.047972	1.045232	1.046975
[70,75)	0.043144	0.045885	0.048753	0.02862	0.030949	0.033265	0.663372	0.674497	0.682317
[75,+)	0.140352	0.145997	0.151866	0.155416	0.164431	0.17482	1.107328	1.126262	1.151142

© 2022 Chun JY et al. *JAMA Network Open.*

eFigure 2. Result of Sensitivity Analysis: 4% Asymptomatic Infections

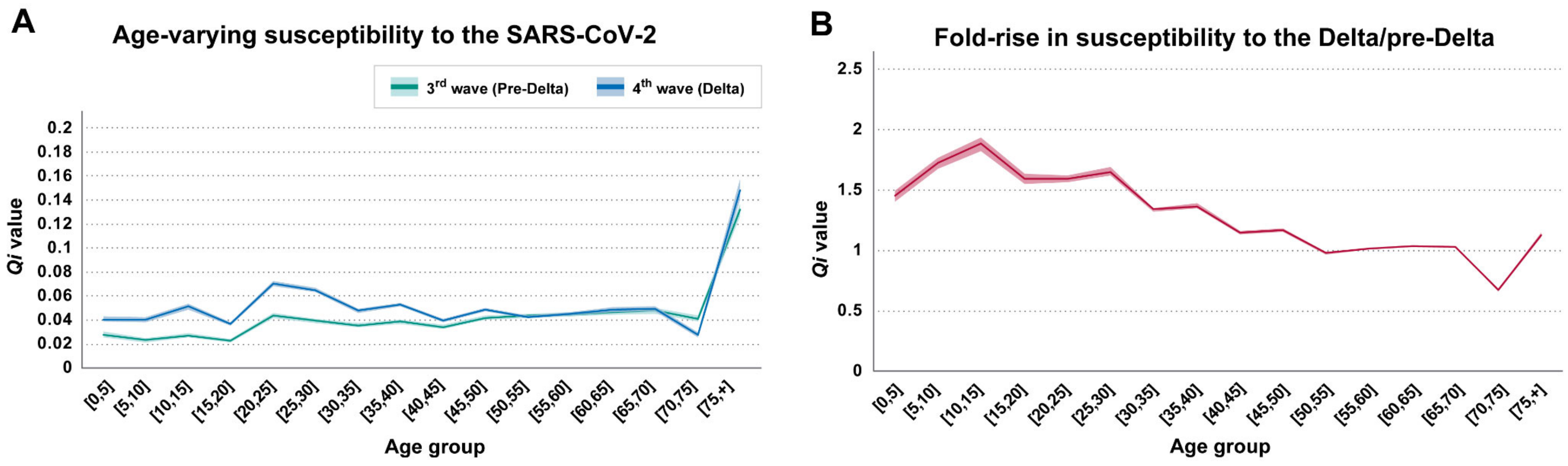

	3rd wave (Pre-Delta)			4th wave (Delta)			Fold-rise		
	2.50%	50%	97.50%	2.50%	50%	97.50%	2.50%	50%	97.50%
[0,5)	0.025865	0.028017	0.030438	0.038677	0.040748	0.042897	1.495301	1.454397	1.409292
[5,10)	0.021731	0.023424	0.025279	0.038392	0.040384	0.042498	1.766694	1.724016	1.681141
[10,15)	0.02539	0.027179	0.02937	0.049117	0.051339	0.053558	1.934465	1.888909	1.823548
[15,20)	0.02161	0.023147	0.024622	0.035323	0.036825	0.038282	1.634593	1.590928	1.554769
[20,25)	0.042101	0.044197	0.04613	0.068304	0.070392	0.072277	1.622399	1.592698	1.566813
[25,30)	0.037596	0.039434	0.041197	0.063482	0.065195	0.06693	1.688559	1.653254	1.624614

© 2022 Chun JY et al. *JAMA Network Open.*

[30,35)	0.034108	0.035655	0.037418	0.046105	0.047775	0.049403	1.351759	1.339918	1.320308
[35,40)	0.037044	0.038699	0.040471	0.051488	0.052922	0.054541	1.389914	1.367557	1.347647
[40,45)	0.032887	0.034472	0.03609	0.038329	0.039628	0.04086	1.165477	1.149573	1.132147
[45,50)	0.040258	0.041917	0.043616	0.047524	0.048936	0.050434	1.180501	1.167439	1.156317
[50,55)	0.042134	0.043743	0.045571	0.041578	0.04286	0.044193	0.986791	0.979801	0.969764
[55,60)	0.04309	0.044721	0.046526	0.043962	0.045336	0.046846	1.020235	1.013743	1.006878
[60,65)	0.044889	0.046779	0.048583	0.046622	0.048595	0.050571	1.038604	1.038817	1.040915
[65,70)	0.045654	0.047994	0.050251	0.047142	0.049538	0.05194	1.032579	1.032169	1.033613
[70,75)	0.039149	0.041289	0.04376	0.02599	0.027916	0.029953	0.663877	0.676113	0.684477
[75+	0.126492	0.13185	0.136889	0.140551	0.148336	0.15708	1.11114	1.125044	1.147502

© 2022 Chun JY et al. *JAMA Network Open.*

eFigure 3. Result of Sensitivity Analysis: 40% Asymptomatic Infections

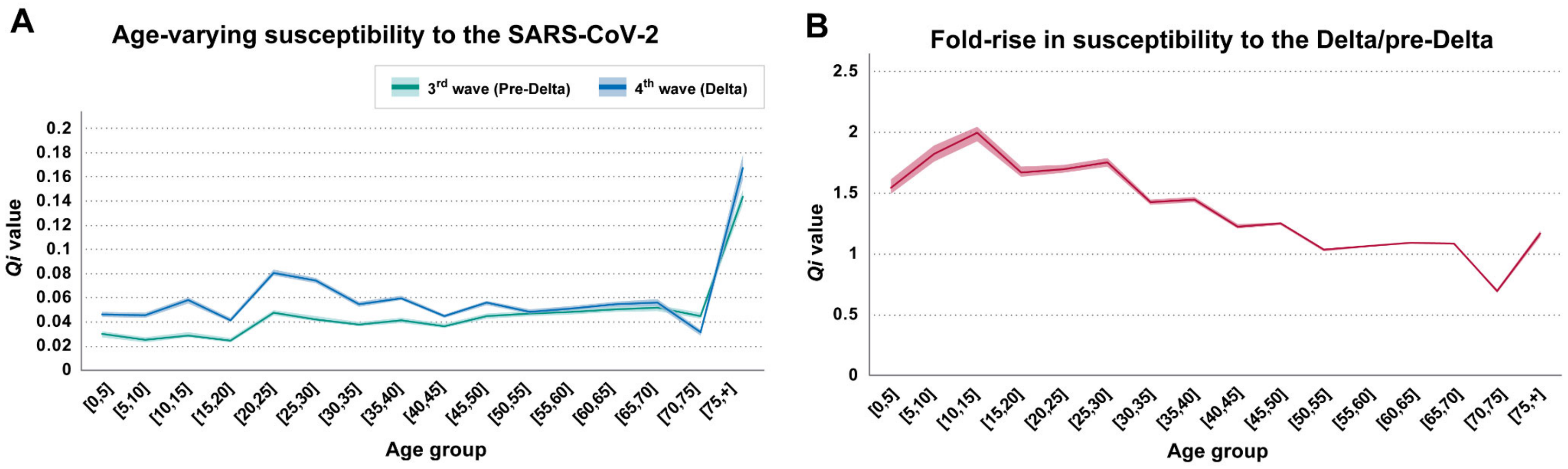

	3rd wave (Pre-Delta)			4th wave (Delta)			Fold-rise		
	2.50%	50%	97.50%	2.50%	50%	97.50%	2.50%	50%	97.50%
[0,5)	0.034055	0.037556	0.040725	0.055123	0.057972	0.060964	1.618664	1.543596	1.496964
[5,10)	0.028982	0.031427	0.034079	0.054844	0.057418	0.060081	1.892356	1.827009	1.762985
[10,15)	0.034077	0.036518	0.039336	0.069659	0.072908	0.075929	2.044187	1.996527	1.930292
[15,20)	0.028991	0.031139	0.033173	0.049922	0.052041	0.054193	1.722011	1.671269	1.633647
[20,25)	0.056732	0.059505	0.062333	0.09836	0.101091	0.10414	1.733764	1.69888	1.670693

© 2022 Chun JY et al. *JAMA Network Open.*

[25,30)	0.050627	0.05319	0.055891	0.090664	0.093327	0.095974	1.790827	1.754605	1.717169
[30,35)	0.045527	0.047774	0.050278	0.065976	0.068175	0.070729	1.449169	1.427031	1.406752
[35,40)	0.049432	0.051736	0.05418	0.072658	0.074838	0.077252	1.469865	1.446546	1.425849
[40,45)	0.043944	0.046094	0.048272	0.054637	0.056456	0.058322	1.243351	1.22481	1.208191
[45,50)	0.05373	0.055979	0.058476	0.067795	0.070014	0.072315	1.261771	1.250725	1.236643
[50,55)	0.05656	0.058938	0.061415	0.059183	0.061022	0.063009	1.046363	1.035359	1.025942
[55,60)	0.058006	0.060313	0.062604	0.06214	0.064275	0.066431	1.071269	1.06569	1.061132
[60,65)	0.060278	0.062956	0.065515	0.065896	0.068645	0.071397	1.093203	1.090351	1.089767
[65,70)	0.061658	0.064603	0.067678	0.066379	0.069972	0.073606	1.076568	1.08311	1.087595
[70,75)	0.053366	0.056472	0.05983	0.036458	0.039311	0.042402	0.683174	0.696119	0.708713
[75+	0.172041	0.17943	0.186494	0.196831	0.209439	0.222033	1.144092	1.167244	1.190564

© 2022 Chun JY et al. *JAMA Network Open.*

eFigure 4. Result of Sensitivity Analysis: Age-Varying Asymptomatic Proportions

52%, 50%, 45%, and 12% among individuals aged 0–4 years, 5–11 years, 12–17 years, and 18 years or older, respectively

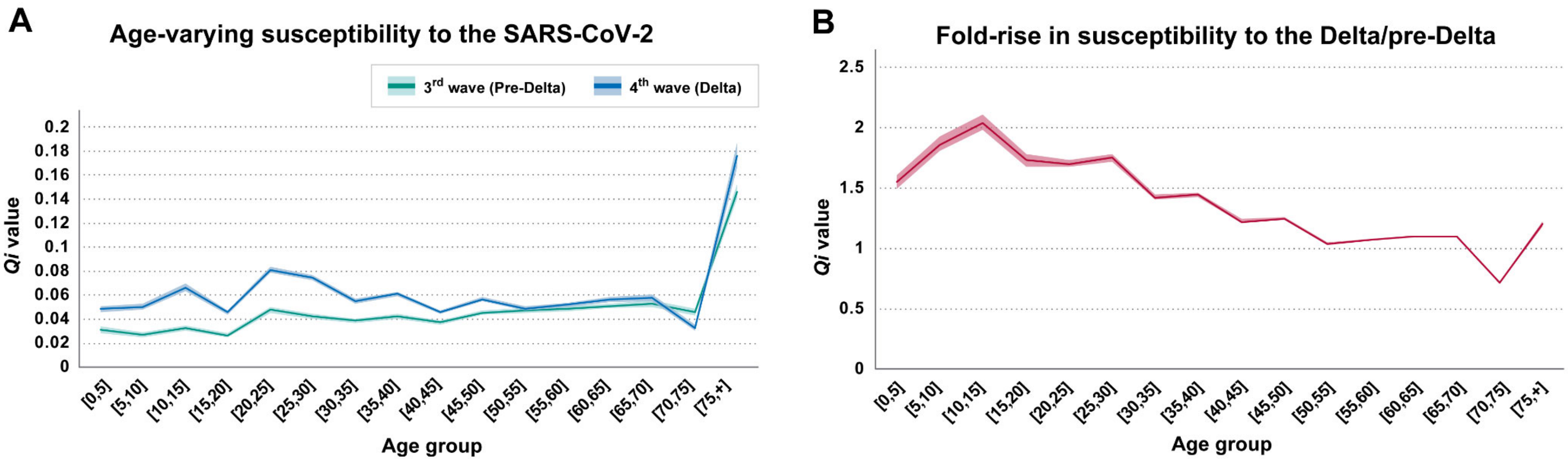

	3rd wave (Pre-Delta)			*4th wave (Delta)*			*Fold-rise*		
	2.50%	50%	97.50%	2.50%	50%	97.50%	2.50%	50%	97.50%
[0,5)	0.028675	0.031236	0.034147	0.046062	0.048522	0.051124	1.606377	1.553394	1.497158
[5,10)	0.024929	0.027085	0.02924	0.048033	0.050374	0.052918	1.926812	1.859873	1.809756
[10,15)	0.030225	0.032527	0.035035	0.063673	0.066354	0.069409	2.106616	2.039944	1.9811
[15,20)	0.024597	0.026322	0.028241	0.043749	0.045612	0.047407	1.778615	1.732831	1.678682
[20,25)	0.045476	0.047701	0.049893	0.078916	0.08111	0.083646	1.735338	1.700378	1.676491

© 2022 Chun JY et al. *JAMA Network Open.*

[25,30)	0.040659	0.042504	0.044425	0.072461	0.074412	0.076394	1.782155	1.750735	1.71961
[30,35)	0.036702	0.03864	0.040541	0.053069	0.054924	0.056863	1.445929	1.421442	1.402599
[35,40)	0.040364	0.042229	0.044234	0.059069	0.060969	0.062992	1.463404	1.443775	1.424069
[40,45)	0.03561	0.037418	0.039101	0.044228	0.045647	0.047333	1.241996	1.219929	1.210533
[45,50)	0.043488	0.045388	0.047337	0.054772	0.056564	0.058474	1.259462	1.246234	1.235264
[50,55)	0.045404	0.047335	0.049215	0.047592	0.049037	0.050564	1.048204	1.035964	1.027417
[55,60)	0.0467	0.048647	0.050525	0.050252	0.051929	0.053586	1.076046	1.067476	1.060567
[60,65)	0.049185	0.051086	0.053098	0.053996	0.056111	0.058203	1.097813	1.098358	1.096151
[65,70)	0.050295	0.052801	0.055437	0.05528	0.058034	0.060712	1.099109	1.09911	1.09515
[70,75)	0.043293	0.045813	0.048555	0.030568	0.032808	0.03522	0.706058	0.71612	0.725355
[75+	0.140277	0.146151	0.152142	0.166294	0.175837	0.186653	1.185464	1.203116	1.226837

© 2022 Chun JY et al. *JAMA Network Open.*

eFigure 5. Result of Sensitivity Analysis: Age-Varying Ascertainment Ratio

1.2 times more cases than reported among those aged < 20 years

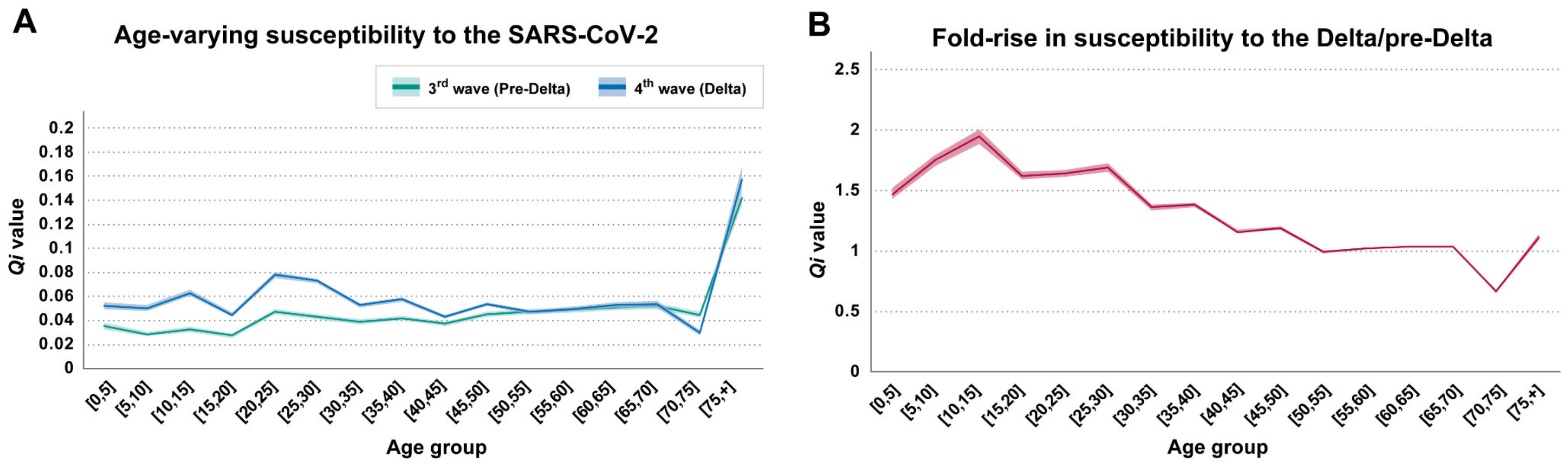

	3rd wave (Pre-Delta)			*4th wave (Delta)*			*Fold-rise*		
	2.50%	50%	97.50%	2.50%	50%	97.50%	2.50%	50%	97.50%
[0,5)	0.032687	0.035518	0.038205	0.049758	0.052199	0.054724	1.522285	1.469626	1.432367
[5,10)	0.026812	0.028739	0.030869	0.048145	0.050386	0.052722	1.79565	1.753225	1.707949
[10,15)	0.030212	0.032356	0.034665	0.060546	0.062999	0.065483	2.00403	1.947042	1.889009
[15,20)	0.025982	0.027553	0.029247	0.043048	0.044681	0.046536	1.656882	1.621632	1.591126
[20,25)	0.04537	0.047496	0.049729	0.075632	0.077844	0.080108	1.667003	1.638964	1.61089

© 2022 Chun JY et al. *JAMA Network Open.*

[25,30)	0.041124	0.043114	0.045105	0.070931	0.072849	0.074815	1.724805	1.689702	1.658671
[30,35)	0.0369	0.038797	0.040823	0.05115	0.052882	0.054547	1.386189	1.363025	1.336186
[35,40)	0.040016	0.04178	0.043692	0.055866	0.0577	0.059538	1.396104	1.381051	1.362693
[40,45)	0.035528	0.037248	0.038774	0.041749	0.043099	0.044656	1.175115	1.157078	1.151718
[45,50)	0.043224	0.045076	0.047013	0.052023	0.053554	0.055167	1.203584	1.18808	1.173448
[50,55)	0.04551	0.047388	0.049275	0.045532	0.046975	0.048462	1.000494	0.991285	0.983507
[55,60)	0.046831	0.048549	0.050566	0.048173	0.049679	0.051223	1.028655	1.023283	1.013
[60,65)	0.04882	0.05092	0.052965	0.0506	0.052817	0.055149	1.036474	1.037249	1.041235
[65,70)	0.049538	0.051932	0.054468	0.05116	0.053685	0.056191	1.032756	1.033754	1.031622
[70,75)	0.042217	0.04471	0.04735	0.0275	0.029682	0.031877	0.651389	0.663883	0.673226
[75+	0.135969	0.141575	0.147052	0.148358	0.157357	0.167277	1.091117	1.111471	1.137537

© 2022 Chun JY et al. *JAMA Network Open.*

eFigure 6. Result of Sensitivity Analysis: 25% Relative Infectiousness of Asymptomatic Infections

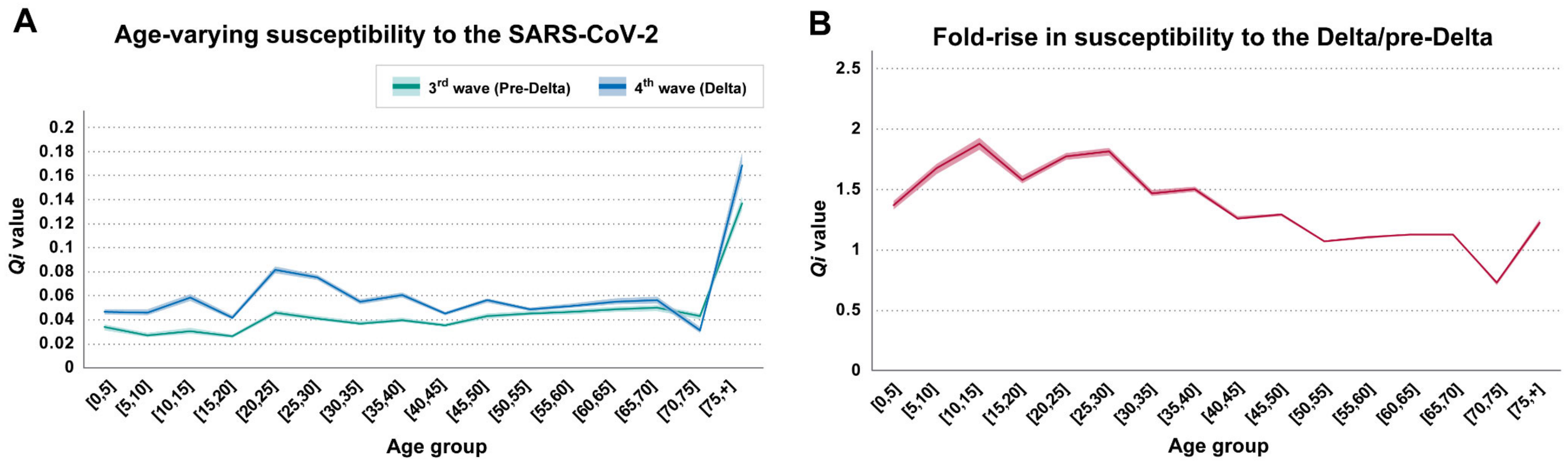

	3rd wave (Pre-Delta)			4th wave (Delta)			Fold-rise		
	2.50%	50%	97.50%	2.50%	50%	97.50%	2.50%	50%	97.50%
[0,5)	0.031706	0.034361	0.036867	0.044636	0.047141	0.049364	1.407799	1.371943	1.338963
[5,10)	0.025844	0.027784	0.029861	0.044438	0.046696	0.048834	1.719482	1.680647	1.635394
[10,15)	0.029204	0.031294	0.033434	0.056383	0.058979	0.061416	1.93068	1.884683	1.836921
[15,20)	0.025104	0.026631	0.028277	0.040576	0.042216	0.043972	1.616337	1.585198	1.555051
[20,25)	0.043937	0.046063	0.048312	0.079526	0.08186	0.084551	1.810027	1.777134	1.750113
[25,30)	0.039841	0.041768	0.043784	0.073777	0.075925	0.078139	1.851758	1.817778	1.784654

© 2022 Chun JY et al. *JAMA Network Open.*

[30,35)	0.035677	0.037524	0.039457	0.053432	0.055333	0.057217	1.497655	1.474604	1.450124
[35,40)	0.038673	0.040417	0.042299	0.059027	0.060872	0.062797	1.526304	1.506096	1.484609
[40,45)	0.034369	0.036001	0.037485	0.044081	0.045584	0.047142	1.282586	1.26617	1.257626
[45,50)	0.041781	0.043604	0.045481	0.054659	0.056543	0.058439	1.308251	1.296741	1.284913
[50,55)	0.044086	0.045892	0.047693	0.047721	0.049323	0.050825	1.082466	1.074755	1.065669
[55,60)	0.045281	0.047015	0.048895	0.050505	0.052143	0.053749	1.115371	1.109077	1.099277
[60,65)	0.047255	0.049264	0.051221	0.053417	0.055757	0.057919	1.130397	1.131802	1.130756
[65,70)	0.047923	0.05029	0.052653	0.054386	0.056885	0.059626	1.134864	1.131146	1.132421
[70,75)	0.041021	0.043412	0.045969	0.029371	0.031768	0.034138	0.716015	0.731784	0.742637
[75+	0.131881	0.137323	0.142714	0.159522	0.168758	0.179414	1.209595	1.22891	1.25716

© 2022 Chun JY et al. *JAMA Network Open.*

eFigure 7. Result of Sensitivity Analysis: 75% Relative Infectiousness of Asymptomatic Infections

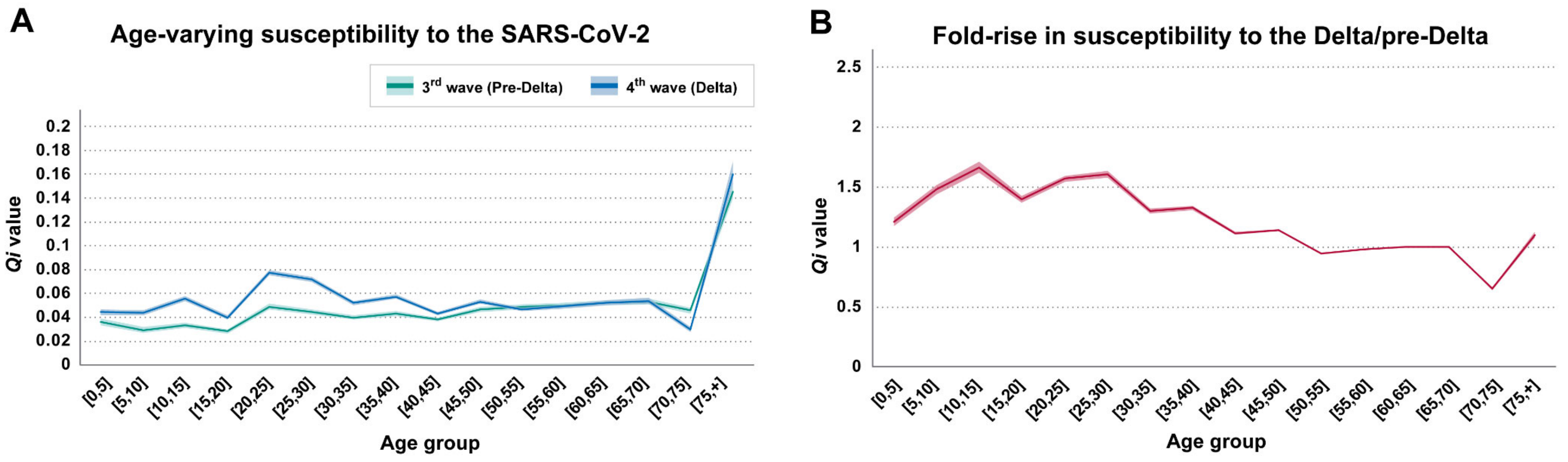

	3rd wave (Pre-Delta)			4th wave (Delta)			Fold-rise		
	2.50%	50%	97.50%	2.50%	50%	97.50%	2.50%	50%	97.50%
[0,5)	0.033943	0.03685	0.039607	0.042358	0.044708	0.046822	1.247914	1.213264	1.182169
[5,10)	0.027768	0.029841	0.032123	0.042186	0.044286	0.046344	1.51926	1.484061	1.442694
[10,15)	0.031355	0.033613	0.035893	0.053688	0.056145	0.058397	1.712256	1.670328	1.62697
[15,20)	0.02704	0.028668	0.030446	0.038613	0.040154	0.041802	1.428026	1.400642	1.373003
[20,25)	0.047153	0.049349	0.051717	0.075459	0.077653	0.080205	1.600281	1.573546	1.55085
[25,30)	0.042717	0.044706	0.046839	0.069965	0.071956	0.07403	1.637871	1.609528	1.580525

© 2022 Chun JY et al. *JAMA Network Open.*

[30,35)	0.038287	0.040287	0.042332	0.05065	0.052459	0.054214	1.322904	1.302129	1.280691
[35,40)	0.041554	0.043426	0.045446	0.055986	0.057748	0.059534	1.347318	1.32981	1.309979
[40,45)	0.036977	0.038701	0.040315	0.041815	0.043246	0.044698	1.130826	1.11743	1.108705
[45,50)	0.044915	0.046807	0.048784	0.051849	0.053657	0.055428	1.154371	1.146347	1.136173
[50,55)	0.047258	0.049217	0.051192	0.045287	0.046786	0.048225	0.958297	0.950619	0.942048
[55,60)	0.048446	0.050282	0.052327	0.047945	0.049476	0.050964	0.989649	0.983977	0.973944
[60,65)	0.050478	0.052637	0.054713	0.050753	0.052908	0.054973	1.005433	1.005161	1.004746
[65,70)	0.051162	0.053621	0.056201	0.051658	0.054016	0.056598	1.009694	1.007365	1.007068
[70,75)	0.043436	0.045993	0.048739	0.027921	0.030186	0.032433	0.642808	0.656301	0.665446
[75+	0.139854	0.145725	0.151337	0.151564	0.16041	0.170559	1.083729	1.100773	1.127015

© 2022 Chun JY et al. *JAMA Network Open.*

eFigure 8. Result of Sensitivity Analysis: Vaccine Efficacy at Lower Bound of 95% CI

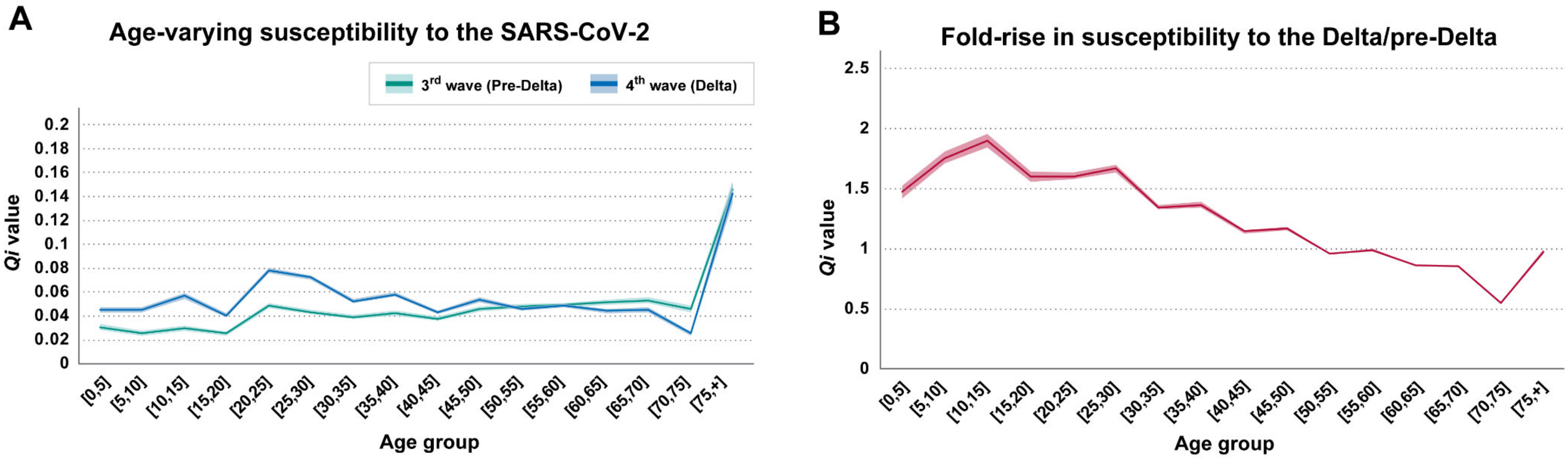

	3rd wave (Pre-Delta)			4th wave (Delta)			Fold-rise		
	2.50%	50%	97.50%	2.50%	50%	97.50%	2.50%	50%	97.50%
[0,5)	0.02829	0.030807	0.033569	0.04305	0.045447	0.047569	1.521747	1.475223	1.417061
[5,10)	0.023685	0.025646	0.027586	0.042912	0.045041	0.047124	1.811787	1.756235	1.708248
[10,15)	0.027847	0.02998	0.032231	0.05455	0.057028	0.059332	1.9589	1.902171	1.840861
[15,20)	0.023795	0.02538	0.027121	0.039022	0.040582	0.042266	1.639939	1.598983	1.558444
[20,25)	0.046352	0.048611	0.051073	0.075683	0.077911	0.080492	1.632785	1.602738	1.576042
[25,30)	0.04139	0.043289	0.045337	0.070158	0.072175	0.074224	1.695051	1.667291	1.637171

© 2022 Chun JY et al. *JAMA Network Open.*

[30,35)	0.037224	0.039097	0.040973	0.050789	0.052562	0.054296	1.364424	1.344406	1.325173
[35,40)	0.040271	0.04238	0.044537	0.056114	0.057846	0.059658	1.393392	1.364952	1.339525
[40,45)	0.036144	0.037729	0.039539	0.041805	0.043217	0.044662	1.156616	1.145471	1.129577
[45,50)	0.043949	0.045958	0.047976	0.051843	0.053621	0.055395	1.179614	1.166747	1.154652
[50,55)	0.04641	0.048158	0.050051	0.044658	0.046113	0.047532	0.962257	0.95753	0.949682
[55,60)	0.047279	0.049315	0.051147	0.047246	0.04877	0.050242	0.99931	0.988937	0.982299
[60,65)	0.049461	0.051628	0.053608	0.042538	0.044387	0.04612	0.860038	0.859746	0.860319
[65,70)	0.050556	0.053013	0.055442	0.043347	0.0453	0.047449	0.857407	0.854512	0.855822
[70,75)	0.043144	0.045885	0.048753	0.023427	0.025309	0.027174	0.543006	0.551571	0.557375
[75+	0.140352	0.145997	0.151866	0.13443	0.142278	0.151246	0.957808	0.974527	0.995918

© 2022 Chun JY et al. *JAMA Network Open.*

eFigure 9. Result of Sensitivity Analysis: Vaccine Efficacy at Upper Bound of 95% CI

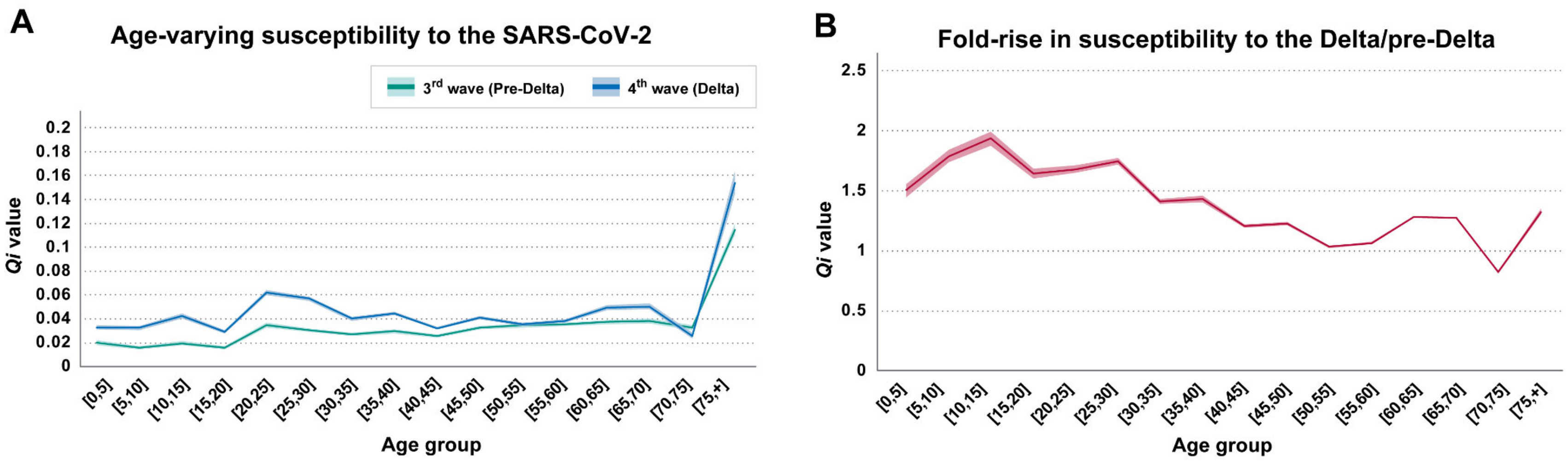

	3rd wave (Pre-Delta)			*4th wave (Delta)*			*Fold-rise*		
	2.50%	50%	97.50%	2.50%	50%	97.50%	2.50%	50%	97.50%
[0,5)	0.02829	0.030807	0.033569	0.043874	0.046316	0.048479	1.550885	1.503437	1.444158
[5,10)	0.023685	0.025646	0.027586	0.043672	0.045865	0.047947	1.843859	1.788349	1.738085
[10,15)	0.027847	0.02998	0.032231	0.055443	0.058013	0.060346	1.990975	1.935038	1.8723
[15,20)	0.023795	0.02538	0.027121	0.040133	0.041716	0.043431	1.68664	1.64365	1.601381
[20,25)	0.046352	0.048611	0.051073	0.079176	0.081483	0.084143	1.708148	1.676218	1.647519
[25,30)	0.04139	0.043289	0.045337	0.073459	0.075573	0.077757	1.77479	1.7458	1.715109

© 2022 Chun JY et al. *JAMA Network Open.*

[30,35)	0.037224	0.039097	0.040973	0.053203	0.055076	0.056962	1.429278	1.408711	1.390225
[35,40)	0.040271	0.04238	0.044537	0.058771	0.06059	0.062526	1.459369	1.429697	1.403918
[40,45)	0.036144	0.037729	0.039539	0.043967	0.045451	0.047015	1.216428	1.204667	1.189095
[45,50)	0.043949	0.045958	0.047976	0.054495	0.056396	0.058266	1.239962	1.227123	1.21449
[50,55)	0.04641	0.048158	0.050051	0.048101	0.04972	0.05126	1.036442	1.032429	1.024154
[55,60)	0.047279	0.049315	0.051147	0.050928	0.052564	0.054145	1.077189	1.065872	1.058608
[60,65)	0.049461	0.051628	0.053608	0.063546	0.066306	0.068849	1.284776	1.28431	1.28431
[65,70)	0.050556	0.053013	0.055442	0.064663	0.067643	0.070902	1.279042	1.275959	1.278847
[70,75)	0.043144	0.045885	0.048753	0.034943	0.037807	0.040606	0.809932	0.823943	0.832892
[75+	0.140352	0.145997	0.151866	0.182573	0.193139	0.205337	1.300821	1.322901	1.352092

© 2022 Chun JY et al. *JAMA Network Open.*
